# Antiprotozoal Activity Against *Entamoeba hystolitica* and *Giardia lamblia* of Cyclopeptides Isolated from *Annona diversifolia* Saff

**DOI:** 10.3390/molecules29235636

**Published:** 2024-11-28

**Authors:** Ulises Murrieta-Dionicio, Fernando Calzada, Elizabeth Barbosa, Miguel Valdés, Benito Reyes-Trejo, Holber Zuleta-Prada, Diana Guerra-Ramírez, Federico del Río-Portilla

**Affiliations:** 1 Laboratorio de Productos Naturales, Área de Química, Departamento de Preparatoria Agrícola, Universidad Autónoma Chapingo, km 38.5 Carretera México-Texcoco, Chapingo 56230, Mexico; murrieta.dionicio.091293@gmail.com (U.M.-D.); hzuletap@chapingo.mx (H.Z.-P.); dguerrar@chapingo.mx (D.G.-R.); 2 Unidad de Investigación Médica en Farmacología, UMAE Hospital de Especialidades, 2° Piso CORSE Centro Médico Nacional Siglo XXI, IMSS, Av. Cuauhtémoc 330, Col. Doctores, Ciudad de México 06725, Mexico; valdesguevaramiguel@gmail.com; 3 Escuela Superior de Medicina, Instituto Politécnico Nacional, Salvador Díaz Mirón esq. Plan de San Luis S/N, Miguel Hidalgo, Casco de Santo Tomas, Ciudad de México 11340, Mexico; rebc78@yahoo.com.mx; 4 Instituto de Química, Universidad Nacional Autónoma de México, Ciudad Universitaria, Mexico City 04510, Mexico; jfrp@unam.mx

**Keywords:** cherimolacyclopeptide D, squamines C and D, cyclopeptides, NMR, molecular docking, antiprotozoal activity, *Entamoeba hystolitica*, *Giardia lamblia*

## Abstract

Diseases caused by intestinal parasites such as protozoa represent a worldwide problem mainly for developing countries. From morbidity in different groups of people to cases of mortality in children and/or immunocompromised patients. In addition to the above, there is growing resistance to the drugs used in the treatment of these diseases, as well as undesirable side effects in patients. Therefore, there is an interest in the search for new alternatives for the base and/or development of new drugs with antiparasitic activities without harmful effects. In this sense, natural products offer to be a diverse source of compounds with biological activities. In this work, we describe the isolation and elucidation by 1D and 2D NMR spectroscopy of three cyclopeptides obtained from seeds of *A. diversifolia* Saff.: cherimolacyclopeptide D (**1**), squamin D (**2**), and squamin C (**3**). The fractions enriched in cyclopeptides, as well as a pure compound (**1**), showed antiprotozoal activity against *E. hystolitica* Schaudinn and *Giardia lamblia* Kunstler in vitro assays, with values of IC_50_ = 3.49 and 5.39 μg mL^−1^, respectively. The molecular docking study revealed that **1** has a strong interaction with targets used, including aldose reductase and PFOR enzymes. The antiprotozoal activity of cherimolacyclopeptide D is reported for the first time in this study.

## 1. Introduction

Intestinal protozoa are responsible for more than one billion intestinal parasitic diseases globally [1]. Due to its level of incidence, amebiasis caused by the protozoan *Entamoeba hystolitica* Schaudinn [2] stands out in importance. Another important case is giardiasis caused by *Giardia lamblia* Kunstler, which causes alterations in intestinal permeability, the microbiota, and the immune system [3]. This contributes to a public health problem such as gastrointestinal diseases, which during the last decade have represented the second cause of morbidity in Mexico [4].

Current therapies are based on the use of synthetic drugs such as nitroimidazoles and/or benzimidazoles, as well as their derivatives [5]. However, these medications can cause undesirable side effects such as nausea, vomiting, abdominal pain, and diarrhea in treated patients [6]. Therefore, there is a need in the search for new bioactive compounds as antiparasitic agents with fewer or no adverse effects. In these contexts, medicinal plants may be an important source of compounds with potential therapeutic effects [7].

*Annona diversifolia* Saff. (*Annona macroprophyllata* Donn), popularly known as “ilama, papauce, lamazapotl, anona blanca, or cherimoya”, is used in Mexican traditional medicine to treat cancer, inflammation, pain, and diabetes mellitus, as well as analgesics. The fruit of this Annonaceae is used as food. Its hexanic and ethanol extracts obtained from the leaves and seeds have been reported with several biological activities such as antinociceptive, anticonvulsant, antimicrobial, antifungal, antitumor, antihyperglycemic, and inhibitor of the α-glucosidase enzyme. From *A. diversifolia* have been isolated acetogenins, flavonoids, alkaloids, and terpenoids. Among the terpenoids isolated by bioassay-guided fractionation were antitumor compounds, geranylgeraniol, farnesyl acetate, and phytol. However, so far there are no scientific studies supporting its potential antiprotozoal activity against *E. histolytica* and *G. lamblia.* In this sense, other species of the genus are known to have those properties, including *Annona cherimola*, *A. muricata*, and *A. squamosa* [8].

Alternative therapies based on the use of organic extracts from medicinal plants have been documented with potential amoebicidal and giardicidal effects [9,10,11,12,13,14]. Recent bio-directed studies on some of these extracts have resulted in the obtaining of natural products such as some flavonoids as kaempferol (CI_50_ = 7.9 and 8.7 μg mL^−1^) [15] or epicatechin (CI_50_ = 1.9 and 1.6 μg mL^−1^) [11] with relevant antiprotozoal activities against *E. hystolitica* and *G. lamblia*, respectively.

An interesting case study is cyclopeptides. Cyclopeptides are peptides of ribosomal origin of cyclic character bound from head to tail that lack disulfide bonds. Structurally, between 5 and 16 amino acid residues are formed. Within their sequence, the dominant presence of glycines and prolines as key amino acids in their macrocyclization mechanism stands out [16]. Cyclopeptides can function as antibiotics, toxins, ion-transport regulators, protein-binding inhibitors, enzyme inhibitors, and immunosuppressants [17]. Also, it has been reported their cytotoxic, anticancer, anti-inflammatory, insecticidal, acaricidal, and antiparasitic activities [17,18,19,20]. Recent studies in the genus *Annona* achieved the isolation and elucidation of four new cyclooctapeptides (squamins C-F) with antiamoebic activity from seeds of *A. globiflora*. Squamins D and F revealed greater antiamoebic activity (CI_50_ = 18.3 and 18.0 μM) compared to the trophozoite stage in *Acanthamoeba castellanii* Neff strains. The highest sensitivity is associated with the *R* conformers of the *S*-oxo-methionine residue [21]. On the other hand, studies on synthetic sulfur derivatives of fexinidazole such as fexinidazole sulfoxide and fexinidazole sulfone also resulted in greater antigiardial and antiamoebic activity compared to the parent drug [22]. The above evidence would point to an important effect on protozoa such as *G. lamblia* and *E. hystolitica* by cyclopeptides with sulfur amino acid residues. However, it is necessary to explain a possible mechanism of action such as that which occurs in the pharmacological activation of metronidazole by enzymes such as pyruvate-ferredoxin oxidoreductase (PFOR) present in the metabolism of anaerobic protozoa [23]. For this purpose, molecular coupling studies are used to confirm the affinity between drugs and enzymes [24].

Therefore, the present work describes the isolation and structural elucidation of cyclopeptides obtained from seeds of *A. diversifolia* Saff. and their evaluation with in silico and in vitro assays of the antiprotozoal activity.

## 2. Results and Discussion

### 2.1. Cherimolacyclopeptide D

**Figure 1:**
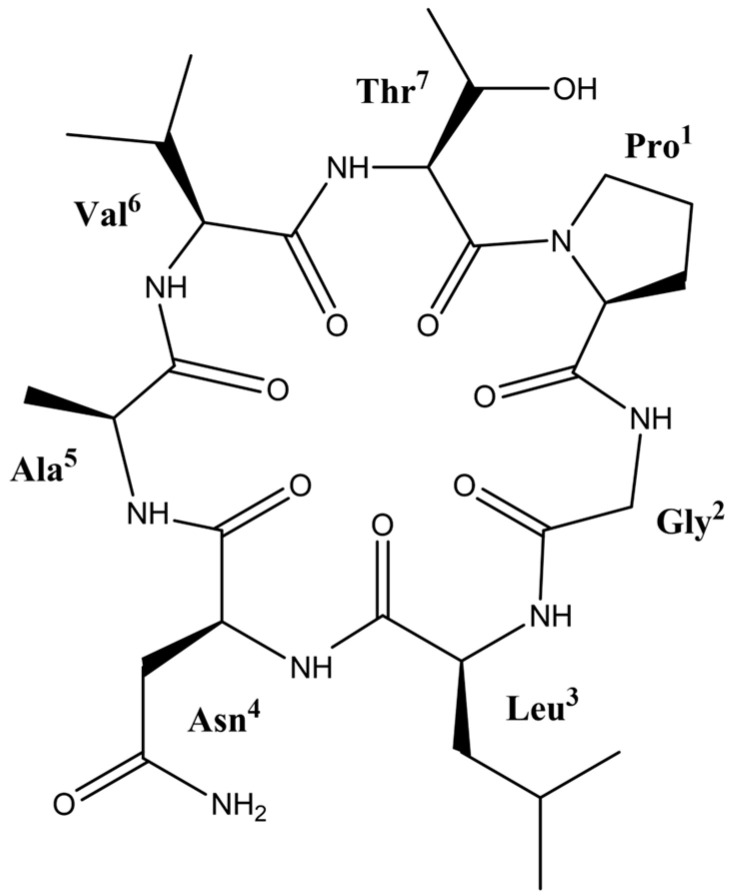
Structure of cherimolacyclopeptide D (**1**) isolated from *A. diversifolia* Saff.

Compound **1**. This compound was obtained as an amorphous white powder; IR λ_max_ cm^−1^ 3304 and 1652 (see Figure S8). NMR data of ^1^H and ^13^C (pyridine-*d*_5_) (see Table 1). MALDI-TOF, *m*/*z*: 675 [M + Na]^+^ (see Figure S7). From the spectroscopy and spectrometry analysis, a calculation was made for a molecular formula of C_29_H_48_N_8_O_9_ corresponding to a molecular weight of 652, with 10 degrees of unsaturation [25]. In the ^1^H-NMR spectrum (see Figure S1 for details), in the range of 8.23–10.36 ppm, six signals corresponding to amide-bonded -NH protons are observed. In addition, two signals at 8.59 and 9.44 ppm correspond to syn and anti-protons of an Asn residue. On the other hand, in the ^13^C-NMR spectrum (see Figure S2 for details) in the region of 169.4–174.7 ppm, signals from eight carbonyl groups are shown. From the HSQC, COSY, and TOCSY spectra (see Figures S3–S5 for details), spin systems corresponding to seven residues of Pro^1^, Gly^2^, Leu^3^, Asn^4^, Ala^5^, Val^6^, and Thr^7^ were assigned (Figure 1). The amino acid sequence was determined with the HMBC spectrum (*J* = 10 Hz) by correlations between the amide proton (-NH) of amino acid *i* and the carbon of the carbonyl group of amino acid *i* + 1. From the HMBC spectrum, six correlations could be assigned between the C=O groups and the -NH groups of amino acid residues. The C=O group at 172.5 ppm of Pro^1^ correlates with the -NH group at 10.37 ppm of Gly^2^. The C=O group at 169.4 ppm of the Gly^2^ correlates with the -NH group at 8.71 ppm of the Leu^3^, the C=O at 172.3 ppm of Leu^3^ correlates with the -NH group at 9.15 ppm of the Asn^4^, the C=O at 171.9 ppm of the Asn^4^ with the -NH at 9.21 ppm of the Ala^5^, the C=O at 173.0 ppm of the Ala^5^ with the -NH at 8.50 ppm of the Val^6^, the C=O at 171.5 ppm of the Val^6^ with the -NH at 8.23 ppm of the Thr^7^. On the other hand, the C=O group at 170.1 ppm of Thr^7^ does not show a correlation with any -NH group in the HMBC spectrum. It follows that this residue is connected to the Pro^1^ residue, thus closing in this manner the cycle (see Figure S6 for details) [26]. The assignment of the second group C=O at 174.7 ppm of the Asn is correlated with the anti at 9.44 ppm and syn at 8.59 ppm protons of the -NH_2_ group. The trans geometry of the Pro was deduced by the γC signal at 24.6 ppm [27]. Finally, based on NMR data and a comparison with literature data, the structure of compound **1** was elucidated as the heptapeptide known as cherimolacyclopeptide D (**1**) [28].

### 2.2. Squamin D

**Figure 2:**
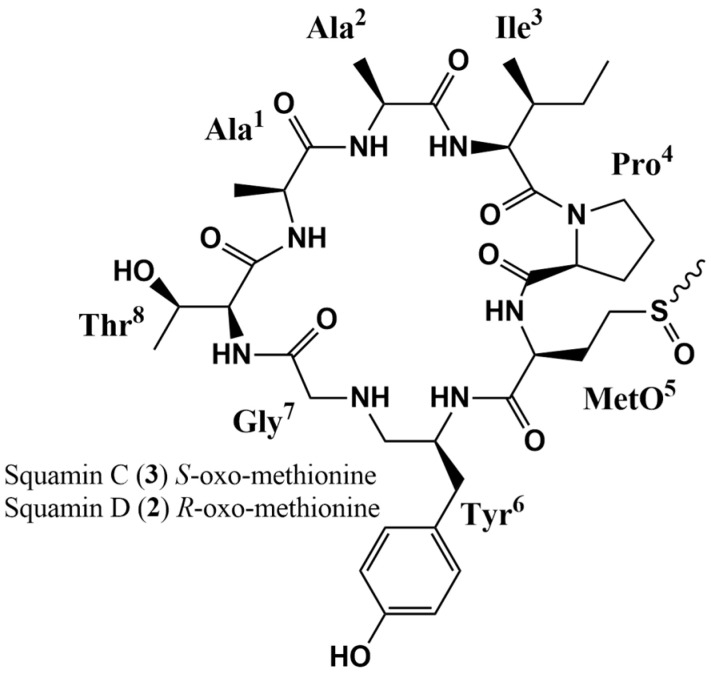
Structures of squamins D (**2**) and C (**3**) isolated from *A. diversifolia* Saff.

Compound **2**. This cyclopeptide was obtained as an amorphous white powder. NMR data of ^1^H and ^13^C (acetone-*d*_6_) (see Table 2). MALDI-TOF, *m*/*z*: 843 [M + Na]^+^ (see Figure S16). From NMR spectra and mass spectrometry, a molecular formula of C_37_H_56_N_8_O_11_S corresponding to a molecular weight of 820 with 14 degrees of unsaturation was determined. In the ^1^H-NMR spectrum (see Figure S9 for details), in the range of 7.08–9.23 ppm, seven characteristic signals for protons of the amide NH type are appreciated. Additionally, in the displacement at 7.30 ppm, there is an integration for two -NH-type protons corresponding to two amino acid residues. From the ^13^C-NMR spectrum in the region of 169.3–175.8 ppm, eight signals are located for carbons of the carbonyl group (C=O) of peptide bonds (see Figure S11 for details). From the number of shifts located in the ^13^C-NMR spectrum, the structure of an octapeptide is suggested (Figure 2). The ^1^H-^1^H assignments of the COSY, TOCSY, and ^1^H-^13^C spectra of the HSQC spectrum (see Figures S12–S14 for details) resulted in the presence of eight amino acid residues, corresponding to the residues of Pro^4^, Met(O)^5^, Tyr^6^, Gly^7^, Thr^8^, Ala^1^, Ala^2^, and Ile^3^ (Figure 2). The Pro^4^ unit was identified by the connections of H_-α_ (*δ*_H_ 4.88, m) with H_2-β_ (*δ*_H_ 1.88/2.27 m/m); in sequence, the union was made with H_2-γ_ (*δ*_H_ 2.27, m) and the latter to H_2-δ_ (*δ*_H_ 3.54/3.71).

With the help of the HMBC spectrum, the assignments for Met(O)^5^ began with H_-α_ (*δ*_H_ 4.05, dt, *J* = 8.3, 4.2 Hz) bound to H_2-β_ (*δ*_H_ 2.39) and sequentially to H_2-γ_ (*δ*_H_ 2.78/2.94). On the other hand, the single methyl group H_3-δ_ (*δ*_H_ 2.65, s) being displaced by a sulfoxide group was assigned by correlation with γC (*δ*_C_ 48.7). For the Tyr^8^ residue, we began with the identification of the spin system corresponding to the disubstituted aromatic ring through the interconnections of the systems H_-δ_/H_-θ_ (*δ*_H_ 6.74, d, *J* = 8.6 Hz) and H_-ε_/H_-η_ (*δ*_H_ 7.05, d, *J* = 8.6 Hz). On the other hand, the spin system of H_-α_ (*δ*_H_ 4.91, m) joined H_2-β_ (*δ*_H_ 2.82/3.63). The interconnection of these two spin systems was carried out with the correlation between H_2-β_ (*δ*_H_ 2.82/3.63) and γC (*δ*_C_ 129.2). Gly was determined with the ^1^H-^1^H couplings between H_2-α_ geminal protons (*δ*_H_ 3.52/4.16, m/dd, *J* = 16.8, 6.7 Hz). The Thr assignment began with H_-α_ (*δ*_H_ 4.96, dd, *J* = 10.0, 3.0 Hz) bound to H_-β_ (*δ*_H_ 4.63, m) and sequentially bound to methyl H_3-γ_ (*δ*_H_ 1.12, d, *J* = 6.3 Hz). In the case of Ala^1^ and Ala^2^, the assignments were made with the connections of the methyl groups H_3-β_ (*δ*_H_ 1.41, d, *J* = 7.4 Hz and *δ*_H_ 1.35, d, *J* = 7.4 Hz, respectively) to the H_-α_ (*δ*_H_ 4.01 dt, *J* = 7.4, 3.7 Hz and *δ*_H_ 4.20 t, *J* = 7.2 Hz, respectively). On the other hand, the Ile residue was assigned starting with the methyl group H_3-δ_ (*δ*_H_ 0.84 t, *J* = 7.5 Hz) joined to H_2-γ_ (*δ*_H_ 1.01/1.44), the next connection was established to H_-β_ (*δ*_H_ 1.99), and this in turn with two more connections, to a methyl group H_3_-ε (*δ*_H_ 0.72 d, *J* = 6.6 Hz) and to H_-α_ (*δ*_H_ 4.37, t, *J* = 10.0 Hz). The sequence of these amino acids was performed with the HMBC spectrum (*J* = 10 Hz). Seven correlations were assigned between the carbonyl groups C=O of the *i* amino acids and the -NH of each amino acid *i* + 1. The C=O group at 175.8 ppm of the Pro^4^ correlates with the -NH group at 9.23 ppm of the Met(O)^5^, the C=O at 170.9 ppm of the Met(O)^5^ with the -NH at 8.00 ppm of the Tyr^6^, the C=O at 171.8 ppm of the Tyr^6^ with the -NH at 8.14 ppm of the Gly^7^, the C=O at 169.3 ppm of the Gly^7^ with the -NH at 7.30 ppm of the Thr^8^, the C=O at 170.9 ppm of the Thr^8^ with the -NH at 7.89 ppm of the Ala^1^, the C=O at 172.6 ppm of the Ala^1^ with the -NH (at 7.30 ppm) of the Ala^2^, the C=O at 172.8 ppm of the Ala^2^ with the -NH at 7.08 ppm of the Ile^3^. In the case of the C=O group of Ile^3^, it has no correlation with any -NH group in the HMBC spectrum, so it can be deduced that the peptide bond binding occurs with the Pro^4^ residue, thus closing the connectivity of the cyclopeptide (see Figure S15 for details). On the other hand, the stereochemistry of *trans*-Pro^4^ was deduced by γC shifts at 24.6 ppm [27]. Finally, based on NMR spectroscopy and comparison with literature data, the structure of compound **2** was elucidated as the octapeptide squamin D (**2**) [29].

### 2.3. Squamin C

Compound **3**. This compound was obtained as an amorphous white powder. The molecular formula obtained for C_37_H_56_N_8_O_11_S matched the same as that determined for compound **2**. NMR data for ^1^H and ^13^C (acetone-*d*_6_) are presented in Table 2. The assignment of signals and correlations was performed with experiments ^1^H-^1^H of the COSY and TOCSY spectra and ^1^H-^13^C of the HSQC and HMBC spectra (see Figures S20–S23 for details), as the description made for compound **2**, establishing a similar structure (Figure 2). The variations in the displacements of both compounds (**2** and **3**) are minimal. However, there is a marked distinction in the signals for the methyl H_3-δ_ (*δ*_H_ 2.65, s) of compound **2** versus H_3-δ_ (*δ*_H_ 2.45, s) for compound **3** (see Figures S9 and S17 for details), both displaced by the presence of a sulfoxide group in the Met(O) residue. In this sense, the determination of the Met-*R*-(O) and Met-*S*-(O) configurations for compounds 2 and 3 was confirmed by a comparison of the ^1^H-NMR data shown in Figures S10 and S18 with those data reported in the literature [29]. The oxidation of the residue from Met to Met (O) generates the introduction of a stereogenic center in the sulfur atom. This implies the formation of two stable diastereomers, Met-*R*-(O) and Met-*S*-(O), since high activation energies are required for there to be an inversion [30]. Finally, based on NMR spectroscopy and comparison with literature data, the structure of compound **3** was deduced as the octapeptide squamin C (**3**), diastereomer of squamin D (**2**) [29].

### 2.4. Antiprotozoal Activity

Based on a general separation procedure for cyclopeptides [27,28,29], a cyclopeptide mixture was obtained by macerating of the seeds with solvents from lowest to highest polarity, in the following order: hexane, CH_2_Cl_2_, EtOAc, acetone, EtOH, MeOH, and an EtOH-H_2_O mixture. Among these, only the extract E-EtOH-H_2_O resulted positive to cyclopeptides presence; therefore, it was suspended with MeOH to obtain a soluble fraction (SF-MeOH) and residue. As the SF-MeOH fraction showed antiprotozoal activity against both protozoans and was positive to the cyclopeptides test, it was fractionated into fractions of different polarity by organic solvent extractions with hexane, CH_2_Cl_2_, EtOAc, and BuOH. All fractions were tested for the cyclopeptides presence and antiprotozoal activity. As the P-BuOH-EM soluble fraction resulted positive to the cyclopeptides test and antiprotozoal activity, it was purified by HPLC to give three cyclodipeptides: cherimolacyclopeptide D (**1**), squamin D (**2**), and squamin C (**3**). All extracts and fractions different from E-EtOH-H_2_O, SF-MeOH, and P-BuOH-EM were discarded because they resulted in negative cyclopeptide presence and showed weak activity against both protozoa [10,11].

The antiprotozoal activity of cyclopeptides **1** and the mixture of **2** and **3** was tested on *E. hystolitica* Schaudinn and *Giardia lamblia* Kunstler. Of these, cherimolacyclopeptide D was the most potent compound against both protozoa, with IC_50_ values of 5. 39 μg mL^−1^ for *G. lamblia* and 3.49 μg mL^−1^ for *Entamoeba hystolitica*. The mixture of **2** and **3** showed selectivity on *Entamoeba hystolitica* (Table 3). To our knowledge, this is the first report of antiamoebic and antigiardial properties of compounds cherimolacyclopeptide D (**1**), squamin D (**2**), and squamin C (**3**). Also, this is the first bioassay-guided work to obtain the antiprotozoal compounds of the seeds of *A. diversifolia*. All compounds were less active than the metronidazole drug used as a positive control [10,11].

### 2.5. Chemoinformatic Analysis of Cherimolacyclopeptide D

Chemoinformatics analysis for the development of new drugs is essential throughout the development process. Various computer tools are used to predict physicochemical and other properties in order to determine whether a molecule is a candidate for developing a new drug. In this sense, we analyzed in silico the physicochemical, pharmacokinetic (ADME), and toxicological properties of cherimolacyclopeptide D (Table 4). In agreement with the informatics results, these suggest that cherimolacyclopeptide D may not be cytotoxic, carcinogenic, or mutagenic. In addition, it does not cause hepatoxicity, neurotoxicity, or cardiotoxicity. Also, the metabolism values indicated that it is a substrate of CYP3A4. On the other hand, it has poor oral absorption and it is not too lipophilic; therefore, it is not a good candidate as an oral drug of systemic action in humans. In contrast, it may be of utility as a drug of local intestinal action like albendazole, for example, in the treatment of diarrhea and dysentery caused by *G. lamblia* and *E. histolytica*, respectively. It also resulted in potential nephrotoxicity, immunotoxicity, and respiratory toxicity. It is classified in class IV and may be fatal for ingestion. In relation to toxic effects, it has been reported that subtle modifications in the chemical structure of cyclopeptides may lead to important changes in the biological activity and toxicity. Considering that cherimolacyclopeptide D exhibited important antiprotozoal activity against *E. histolytica* and *G. lamblia* but several toxic effects, it is an excellent candidate to make changes in its chemical structure and obtain the best activity free of toxic effects [31,32].

### 2.6. Molecular Coupling Studies of Cherimolacyclopeptide D and Fidalrestat on Aldose Reductase

In order to determine the possible mechanism of action of cherimolacyclopeptide D, a molecular coupling was carried out using the enzyme aldose reductase as a target; this enzyme has been reported as a potential target in protozoa such as *E. histolytica* and *G. lamblia*.

Molecular coupling analysis showed that cherimolacyclopeptide D (**1**) has an affinity for the enzyme aldose reductase with ΔG values of −5.94 Kcal-mol^−1^, showed ten polar interactions with amino acid residues Gln13 (3.3 Å) and Trp307 (2.7 Å), and six nonpolar interactions. With respect to fidalrestat, an aldose reductase inhibitor, it showed affinity values of −6.29 Kcal-mol^−1^, with polar interactions on Trp307 (3.3 Å) and Cys301 (2.4 Å), and eight nonpolar interactions (Table 5).

**Figure 3:**
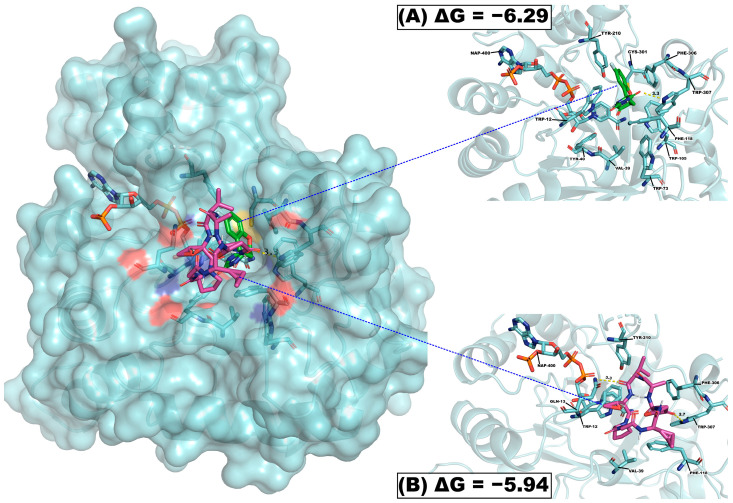
Results of molecular coupling of the enzyme aldose reductase. (**A**) Interaction of the fidalrestat and its position at the attachment site; (**B**) interaction of cherimolacyclopeptide D and its position at the binding site.

Analysis of the 3D binding model suggests that the ligands of cherimolacyclopeptide D have the same binding pocket, and their position is similar to that of fidalrestat (Figure 3). It is important to note that the amino acid portions 303–311 are important for the activity of aldose reductase. Although the ΔG values obtained from cherimolacyclopeptide D were lower than those of fidalrestat, we do not rule out the possible inhibition of aldose reductase. In this sense, it has been reported that ΔG values lower than the threshold of ≤5.0 Kcal-mol^−1^ suggest that the compound forms a stable binding conformation with the target protein [33]. The ΔG between cherimolacyclopeptide D and aldose reductase was lower than the threshold, suggesting that it could easily form a stable binding conformation. This result supports the idea that cherimolacyclopeptide D could inhibit aldose reductase and its therapeutic potential for the treatment of diarrhea and dysentery caused by *Giardia lamblia* and *Entamoeba hystolitica*, respectively.

### 2.7. Molecular Coupling Studies of Cherimolacyclopeptide D and Metronidazole on Ferredoxin Pyruvate Oxidoreductase (PFOR)

The other molecular coupling study was conducted using the pyruvate enzyme ferredoxin oxidoreductase (PFOR) with metronidazole as the control ligand.

**Figure 4:**
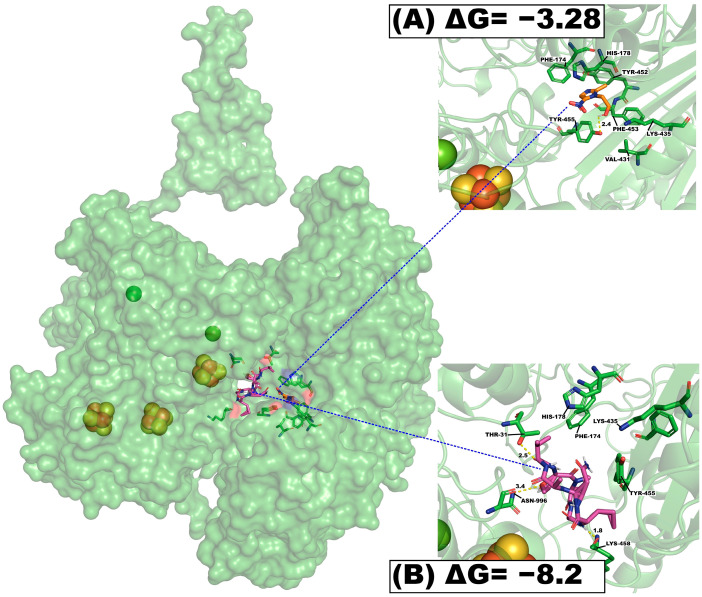
Results of molecular coupling of the enzyme pyruvate ferredoxin oxidoreductase. (**A**) Interaction of metronidazole and its position at the binding site; (**B**) interaction of cherimolacyclopeptide D and its position at the binding site.

The analysis of the molecular coupling results showed that cherimolacyclopeptide D has the best free binding energy (ΔG) values of −8.2 Kcal-mol^−1^, with three polar interactions Thr31 (2.5 Å), Asn996 (3.4 Å), Lys458 (1.8 Å), and six nonpolar interactions with PFOR amino acid residues; this score was higher than that of the PFOR inhibitor metronidazole. The latter showed ΔG values of −3.28 Kcal-mol^−1^, with only one polar interaction with Tyr455 (2.4 Å) and six nonpolar interactions (Table 6). Analysis of in silico interactions, calculated ΔG values, and 3D conformation of the binding site suggest that cherimolacyclopeptide D has a better affinity for PFOR than metronidazole at the same binding position (Figure 4).

The result suggests that cherimolacyclopeptide D has the potential of forming a stable binding conformation with the PFOR; therefore, this is a potential target of its cyclopeptide [33]. Also, this result supports its therapeutic potential for the treatment of diarrhea and dysentery caused by *Giardia lamblia* and *Entamoeba hystolitica*, respectively.

## 3. Materials and Methods

### 3.1. General Experimental Procedure

IR spectra were obtained in a Cary 630 FTIR spectrometer (Agilent^®^, Santa Clara, CA, USA). Thin layer chromatography (TLC) was performed on silica gel plates 60 F_254_ (Merck^®^, Darmstadt, Germany) developed with Cl_2_/*o*-tolidine reagent (Sigma Aldrich, St. Louis, MO, USA). The RP-HPLC separation was performed in a Waters HPLC system (Milford, MA, USA) equipped with a 2535 quaternary pump, connected to a 2707 autosampler, and two PDA 2998 and ELSD 2424 detectors. Data acquisition and processing were performed with Empower 3 (Waters) software. The columns used were C18 Gemini-NX particle size of 5 μm (4.6 mm i.d. × 250 mm and 10.0 mm i.d. × 250 mm for analytical and semi-preparatory experiments, respectively, Phenomenex, Torrance, CA, USA). The ^1^H and ^13^C NMR spectra were acquired in a Bruker ASCEND-700 spectrometer (Bruker BioSpin, Billerica, MA, USA) equipped with a Bruker 5 mm TCI CryoProbe probe at 300 K. The 1D and 2D NMR spectra were acquired in acetone-*d*_6_, CD_3_OD, or pyridine-*d*_5_ (Sigma-Aldrich, St. Louis, MO, USA) at 298 K. Pulse sequences and phase cycles were used for the COSY, HSQC, and HMBC spectra. NMR data were processed using MestReNova 15.0.1 software (Mestrelab Research SL, San Diego, CA, USA).

### 3.2. Vegetal Material

The fruits of *A. diversifolia* Saff. were acquired in the municipal market of Plaza de Gallos, Tejupilco, State of Mexico (18°51′05″ N and 100°14′38″ W), in October 2019. Fruits harvested with ripeness for consumption were selected according to the cracking of the peel in the peri-peduncular zone. The fruit was botanically authenticated, and a specimen (Voucher 36509) was deposited at the Herbarium Jorge Espinosa Salas of the Universidad Autónoma Chapingo (UACh).

### 3.3. Extraction and Isolation

The seeds were manually separated from the fruit and washed with running water in order to remove traces of mucilage and/or pulp. They were then dried at room temperature for two weeks. Seeds that rot or cause insect damage were removed. The almonds (endosperm) were separated from the coriaceous endocarp with the help of mechanical tweezers and then ground in a food processor (NutriBullet^®^, NBR-0804B, Los Angeles, CA, USA). The almonds of *A. diversifolia* Saff. (2.0 kg) were macerated at room temperature (3 × 4 L × 3 days) with solvents from lowest to highest polarity, in the following order: hexane, CH_2_Cl_2_, EtOAc, acetone, EtOH, MeOH, and an EtOH-H_2_O mixture (1:1 *v*/*v*, 0.1% acetic acid) [34]. The solvents were removed in each maceration by reduced pressure evaporation using a Rotavapor R-300 rotary evaporator (Büchi^®^, Meierseggstrasse, Flawil, Switzerland) and reused in the following maceration, finally obtaining seven extracts: hexane (403 g), CH_2_Cl_2_ (439 g), EtOAc (43 g), acetone (17 g), EtOH (6 g), MeOH (23 g), and EtOH-H_2_O (73 g).

The presence of cyclopeptides in each extract was monitored by TLC on 60 F_254_ silica gel plates (Merck^®^, Darmstad, Germany) with CH_2_Cl_2_-MeOH (85:15) as the elution system and as the Cl_2_/*o*-tolidine developer (Sigma Aldrich, St. Louis, MO, USA) [35]. The hydroethanolic extract showed a positive reaction with the Cl_2_/*o*-tolidine developer for the detection of cyclopeptides, so it underwent a purification process.

A total of 42.41 g of hydroethanolic extract was taken and dissolved in 100% MeOH. The soluble fraction (SF-MeOH; 31.32 g) was concentrated at reduced pressure using a Rotavapor R-300 rotary evaporator (Büchi^®^, Meierseggstrasse, Flawil, Switzerland). Subsequently, the fraction was solubilized into 500 mL of H_2_O and subjected to a series of successive liquid–liquid extractions using the solvents Hexane, CH_2_Cl_2_, EtOAc, and BuOH (3 × 0.5 L, 1:1) [36]. From the BuOH partition, a viscous brown residue (2.39 g) was obtained. The residue was analyzed using a Biotage IsoleraTM one ultrafast chromatography (C1) system (Biotage, Uppsala, Sweden) equipped with a Biotage^®^ SNAP ULTRA C18 30 g reversed-phase cartridge (A = H_2_O, B = ACN; 0% B 7 VC, 0–10% B 5 VC, 10–15% B 7 VC, 15–20% B 7 VC, 20–100% B 5 VC, 100% B 4VC) [37]. A total of 86 fractions were obtained, which were concentrated and monitored with the reaction of Cl_2_/*o*-tolidine in TLC. The group of fractions C1-F:49–69 (303.0 mg) was positive for the reaction with Cl_2_/*o*-tolidine, so it was purified with column chromatography (C2) with Kieselgel 60H silica gel (Merck^®^, Darmstadt, Germany), with a CH_2_Cl_2_-MeOH elution system with increments of 5% in 5% to 20% MeOH. Next, 62 fractions of 25 mL were collected, concentrated and monitored with TLC, and finally grouped with similar RFs. The C2-F:25–33 fractions (159.4 mg) tested positive for the chemical reaction of cyclopeptides (Cl_2_/*o*-tolidine). The C2-F:25–33 gather was dissolved in 100% H_2_O and subsequently purified in a solid-phase extraction (SPE) (C3) system. A J.T. Baker^®^ BAKER SPE-12GTM vacuum manifold (Phillipsburg, NJ, USA) was used. It was equipped with STRATA-X^®^ PRO reversed-phase polymer cartridges, 500 mg 6 mL^−1^ (Phenomenex^®^, Torrance, CA, USA).

The cartridges were pre-conditioned with 6 mL of ACN followed by 6 mL of H_2_O. After loading the sample, the cartridges were washed three times with H_2_O-ACN gradients (100:0, 90:10, 80:20, 70:30, and 0:100, *v*/*v*) with 0.05% trifluoroacetic acid (TFA) [38]. A total of 15 subfractions were obtained. The C3-SPE: IV subfraction (26.7 mg) was finally taken to high-performance liquid chromatography (HPLC) equipment. For the separation and/or purification of analytes contained in the sample, a semi-preparative liquid chromatography was used where 500 μL of F:25-33-SPE:IV sample was repeatedly injected at a concentration of 15 mg mL^−1^ into a Waters HPLC equipment with a linear elution gradient of H_2_O (A; 0.05 % TFA *v*/*v*)-ACN (B; 0.05% TFA *v*/*v*) starting from 20 to 35% of B for 30 min at a flow rate of 4.6 mL min^−1^ at 25 °C. The detection of the compound was performed with UV monitoring at lengths of 200, 220, and 280 nm [39]. The process produced three cyclopeptides called cherimolacyclopeptide D (**1**), squamin C (**2**), and D (**3**). Structural elucidation of the isolated compounds was performed by one- and two-dimensional NMR spectroscopy and matrix-assisted laser ionization/desorption time-of-flight mass spectrometry (MALDI-TOF).

### 3.4. NMR Spectroscopy

The purified compounds were dissolved in 0.6 mL of acetone-*d*_6_, CD_3_OD, or pyridine-*d*_5_. Tetramethylsilane (TMS) (Sigma-Aldrich, St. Louis, MO, USA) was used as an internal reference. Dissolved samples were transferred to 3 mm Norrell^®^ Sample Vault Series™ glass tubes (Norell, Inc., Morganton, NC, USA). NMR experiments were performed on a Bruker ASCEND-700 spectrometer. One-dimensional spectra of ^1^H-, ^13^C- NMR and two-dimensional spectra of HSQC, HMBC (*J* = 10 Hz), TOCSY (80 ms, mixing time), and NOESY (300 ms, mixing time) were acquired.

### 3.5. Mass Spectrometry

The molecular mass of the purified cyclopeptides was obtained by MALDI-TOF experiments on a Bruker Daltonics Microflex LT (Bruker Daltonics, Billerica, MA, USA). For its study, 100 nmol of each cyclopeptide was mixed with 3 μL of a matrix (a supersaturated solution of α-cyano-4-hydroxycinnamic acid in ACN 65% in H_2_O added with TFA at 0.05% *v*/*v*). Of this mixture, 1 μL was taken and placed on the MALDI plate for analysis. Data were acquired in a m/z range of 0–1000 Da. The searchlight mode of operation was used by acquiring 200 rounds [40].

### 3.6. Antiprotozoal Tests

*Entamoeba histolytica* strain HM1-IMSS used in all experiments was grown axenically at 37 °C in TYI-S-33 medium supplemented with 10% heat-inactivated bovine serum. In the case of *Giardia lamblia*, strain WB was grown in TYI-S-33 modified medium supplemented with 10% calf serum, bovine bile, and penicillin-streptomycin (0.1%, Gibco). When the logarithmic phase of growth was reached, the trophozoites were detached from the medium and used. Antibiotics were omitted during experimental assays. In vitro susceptibility tests were performed using *E. histolytica* (6 × 10^3^) or *G. lamblia* (5 × 10^4^) trophozoites that were incubated for 48 h at 37 °C in the presence of different concentrations (1.25–200 µg mL^−1^) of the crude extract, fraction, or pure compounds in DMSO at 2%. Each test included metronidazole as a standard amoebicidal and giardicidal drug, a control (culture medium plus trophozoites and DMSO), and a blank (culture medium). After incubation, the trophozoites were detached by chilling, and 50 µL samples of each tube were subcultured in a fresh medium for another 48 h. The final number of parasites was determined with a hemocytometer. Then, data were analyzed using probit analysis. The percentage of trophozoites surviving was calculated by comparison with the growth in the control group. The plot of probit against log concentration was made, the best straight line was determined by regression analysis, and the IC_50_ values were calculated. The regression coefficient, its level of significance (*p* < 0.05 indicates a significant difference between groups), and correlation coefficient were calculated, and 95% confidence interval (CI) values were determined. Results were analyzed using GraphPad Prism version 5.0 (San Diego, CA, USA) software. Performing one-way ANOVA and performing multiple comparison tests using Bonferroni’s test.

### 3.7. Molecular Docking Studies

The chemical structure of the ligand’s metronidazole (CID: 445070) and fidarestat (CID: 160024) were retrieved from the PubChem chemical library (https://pubchem.ncbi.nlm.nih.gov/, accessed 15 July 2024), optimized, and subjected to energy and geometric minimization using Avogadro V1.2.0 [41,42] software. The objective of the study was pyruvate ferredoxin oxidoreductase (crystal structure of the pyruvate intermediate free radical: ferredoxin oxidoreductase, RCSB, PDB ID: 1KEK) and aldose reductase (structure of the aldose reductase of Giardia lamblia, RCSB, PDB ID: 3KRB). They were retrieved from the Protein Data Bank (http://www.rcsb.org/, accessed 15 July 2024). In the case of PFOR, all water molecules not necessary for catalytic activity were removed, and thiamine ions and di-phosphate were maintained while preserving the catalytic activity of the enzyme. As for aldose reductase, all water molecules were removed, as well as ions and small molecules, and the cofactor nicotinamide was retained to preserve the protein.

In both proteins, all polar hydrogen atoms were added, ionized in a basic environment (pH = 7.4), and assigned Gasteiger charges. The output topologies calculated from the previous steps were used as input files for the coupling simulations.

The molecular coupling experiments were carried out using Autodock 4.2 software [42]. The search parameters were as follows: A grid-based procedure was used to generate the affinity maps by delimiting a grid box of 126 × 126 × 126 Å^3^ at each spatial coordinate, with a spacing between the points of the grid of 0.375 Å. Lamarck’s genetic algorithm was used as a scoring function with a random initial population of 100 individuals and a maximum number of energy assessments of 1 × 10^7^ cycles. The analysis of interactions in the enzyme/inhibitor complex was visualized with the PyMOL software (The PyMOL Molecular Graphics System, Ver 2.0, Schrödinger, LLC, DeLano Scientific, San Carlos, CA, USA).

The validation of the molecular coupling was carried out by recoupling the cocrystallized ligand into the proteins (PFOR and aldose reductase). The lowest energy pose of the cocrystallized ligand was superimposed and observed if it maintained the same bond position. The RMSD was calculated, and a reliable range of 2 Å was obtained.

### 3.8. In Silico Physicochemical, Pharmacokinetic, and Toxicological Properties

To determine the physicochemical, pharmacokinetic, and toxicological properties, the SwissADME [43], admetSAR [44], and PROTOX [45] servers were used. The cherimolacyclopeptide D was drawn in the ChemDraw^®^ software V22.0.0.22, and then the SMILES code was obtained, and this code was used in all servers to predict the properties.

## 4. Conclusions

The present phytochemical research on seeds of *A. diversifolia* Saff. achieved the isolation and structural elution of three cyclopeptides. Fractions enriched in cyclopeptides as well as a pure compound (**1**) (cherimolacyclopeptide D) showed antiprotozoal activity against *Entamoeba hystolitica* and *Giardia lamblia*. The results in silico and molecular docking are consistent with those presented in vitro. Compound (**1**) may be a candidate as a potential antiamoebic agent.

## Figures and Tables

**Table 1:** ^1^H and ^13^C NMR (700 MHz) data of cherimolacyclopeptide D (**1**) in pyridine-*d*_5_.

Aminoacid	Position	*δ*_C_	*δ*_H_, mult. (*J* in Hz)
Pro^1^	CO	172.5	
	αCH	61.6	4.42, dd (9.0, 7.2)
	βCH_2_	29.4	1.93, m 2.01, m
	γCH_2_	24.6	1.34, q (10.3) 1.71, dp (10.1, 3.4)
	δCH_2_	48.4	3.62, td (9.7, 6.8) 4.01, m
Gly^2^	CO	169.4	
	NH		10.36, dd (8.4, 4.5)
	αCH_2_	43.6	3.84, dd (17.0, 4.4) 4.78, dd (17.0, 8.3)
Leu^3^	CO	172.2	
	NH		8.71, d (10.22)
	αCH	54.1	5.40, m
	βCH_2_	44.3	1.76, ddd (13.1, 8.7, 6.8) 1.89, t (6.9)
	γCH	24.8	1.92, m
	δCH_3_	22.2	0.88, d (6.5)
	δ′CH_3_	22.3	0.90, d (6.3)
Asn^4^	CO	171.9	
	NH		9.15, d (5.2)
	αCH	51.2	5.06, td (4.8, 2.9)
	βCH_2_	35.8	3.76, m 3.92, dd (16.2, 4.8)
	NH		8.59, s
	NH		9.44, s
	CO	174.7	
Ala^5^	CO	173.0	
	NH		9.21, d (3.7)
	αCH	52.9	4.62, dp (7.5, 3.6)
	βCH_3_	17.4	1.49, d (7.4)
Val^6^	CO	171.5	
	NH		8.49, d (10.3)
	αCH	59.0	5.20, dd (10.4, 6.1)
	βCH	30.8	2.79, sextete (6.5)
	γCH_3_	18.1	1.08, d (6.9)
	γ′CH_3_	19.5	1.08, d (6.9)
Thr^7^	CO	170.1	
	NH		8.23, d (9.5)
	αCH	57.5	5.32, t (9.3)
	βCH	68.1	4.45, dq (8.9, 6.2)
	γCH_3_	20.0	1.57, d (6.2)

**Table 2:** ^1^H and ^13^C NMR data for squamins D (**2**) and C (**3**) in acetone-*d*_6_.

Aminoacid	Position	Squamin D (2)		Squamin C (3)	
		*δ*_C_	*δ*_H_, multi. (*J* in Hz)	*δ*_C_	*δ*_H_, multi. (*J* in Hz)
Ala^1^	CO	172.6		172.6	
	NH		7.89, d (3.9)		7.91, d (3.8)
	αCH	52.0	4.01, dt (7.4, 3.7)	52.0	4.01, qd (7.4, 3.8)
	βCH_3_	15.8	1.41, d (7.4)	15.8	1.41, d (7.4)
Ala^2^	CO	172.8		172.8	
	NH		7.30, d (10.0)		7.29, d (3.8)
	αCH	50.7	4.20, t (7.2)	50.7	4.20, t (7.2)
	βCH_3_	17.1	1.35, d (7.4)	17.1	1.35, d (7.4)
Ile^3^	CO	171.8		171.9	
	NH		7.08, d (9.7)		7.09, d (9.6)
	αCH	54.8	4.37, t (10.0)	54.9	4.36, t (10.0)
	βCH	35.9	1.99, m	35.9	2.01, m
	γCH_2_	23.8	1.01, ddd (13.7, 9.8, 7.4) 1.44, m	23.7	0.99, m 1.44, m
	δCH_3_	10.7	0.84, t (7.5)	10.7	0.83, t (7.5)
	εCH_3_	16.7	0.72, d (6.6)	17.4	0.77, d (6.6)
Pro^4^	CO	175.8		175.7	
	αCH	63.1	4.88, m	63.1	4.89, m
	βCH_2_	29.4	1.88, m 2.27, m	29.5	1.86, dtd (12.3, 9.4, 6.9) 2.27, ddt (12.3, 7.9, 5.2)
	γCH_2_	24.6	2.00, m	24.6	1.99, m
	δCH_2_	47.4	3.54, dd (6.1, 3.8) 3.71, dt (10.0, 7.6)	47.4	3.56, m3.68, m
Met(O)^5^	CO	170.9		170.9	
	NH		9.23, d (3.9)		9.06, d (4.2)
	Ach	55.1	4.05, dt (8.3, 4.2)	55.1	4.06, dt (8.7, 4.4)
	βCH_2_	24.3	2.39, dddd (15.6, 9.7, 4.2, 3.2)	23.1	2.19, m
	γCH_2_	48.7	2.78, m 2.94, ddd (14.4, 9.6, 3.3)	48.0	2.74, ddd (14.5, 8.4, 4.0) 2.99, ddd (14.7, 7.9, 4.0)
	δCH_3_	36.9	2.65, s	35.8	2.45, s
Tyr^6^	CO	171.8		171.8	
	NH		8.00, d (10.0)		8.01, d (10.0)
	αCH	52.1	4.91, m	52.2	4.90, dd (9.5, 2.5)
	βCH_2_	35.9	2.82, d (3.7) 3.63, d (15.8, 3.0)	35.9	2.83, m 3.63, dd (15.8, 3.1)
	γC	129.2		129.2	
	δCH/θCH	114.9	6.74, d (8.6)	114.9	6.76, d (8.6)
	εCH/ηCH	128.9	7.05, d (8.6)	129.0	7.07, d (8.6)
	ζC	155.6		155.7	
Gly^7^	CO	169.3		169.3	
	NH		8.14, t (6.3)		8.09, t (6.2)
	αCH_2_	43.4	3.52, m 4.16, dd (16.8, 6.7)	43.4	3.52, dd (16.8, 5.9) 4.17, dd (16.7, 6.7)
Thr^8^	CO	170.9		170.9	
	NH		7.30, d (10.0)		7.30, d (6.8)
	αCH	55.6	4.96, dd (10.0, 3.0)	55.6	4.96, dd (10.0, 3.0)
	βCH	69.7	4.63, m	69.7	4.63, m
	γCH_3_	19.1	1.12, d (6.3)	19.1	1.12, d (6.3)

**Table 3:** In vitro antiprotozoal activity of extracts, fractions, and pure compounds.

	IC_50_ μg mL^−1^ (CI) ^a^	
Extract/Partition/Fraction/Compound	*Giardia lamblia*	*Entamoeba hystolitica*
E-EtOH-H_2_O	213.01 (213.41–212.60)	67.90 (68.31–67.48)
SF-MeOH	58.23 (58.27–58.19)	49.90 (49.19–48.85)
P-BuOH-EM	20.99 (21.01–20.97)	34.77 (34.91–34.64)
FE-Cherimolacyclopeptide D	53.08 (53.13–53.04)	0.82 (0.85–0.80)
FE-Squamin C (**3**) + Squamin D (**2**)	52.93 (52.96–52.89)	8.68 (8.71–8.64)
Cherimolacyclopeptide D (**1**)	5.39 (5.40–52.38)	3.49 (3.50–3.48)
Metronidazole	0.41 (0.42–0.40)	0.04 (0.10-0.03)

^a^ Results are expressed as a mean (*n* = 6), CI, 95% confidence intervals, and correlation coefficient > 0.900.

**Table 4:** Expected physicochemical, pharmacokinetic, and toxicological properties for cherimolacyclopeptide D (**1**).

Smiles	O=C1N[C@@H]([C@@H](O)C) C(N2[C@@H](CCC2) C(NCC(N[C@@H](CC(C)C)C(N[C@@H](CC(N)=O)C(N[C@@H](C)C(N[C@H]1C(C)C)=O)=O)=O)=O)=O)=O		
Physicochemical properties			
Molecular formula	C_29_H_48_N_8_O_9_	Druglikeness	
Molecular weight	652.74 g/mol	Lipinsky	No, 3 violations
TPSA	258.23 Å	Ghose	No, 4 violations
Lipophilicity (LogP)	−2.09	Veber	No, 1 violation
Water solubility (LogS)	−3.50	Egan	No, 1 violation
Solubility class	Soluble	Muegge	No, 1 violation
Number of rotating links	6		
Number of H-bond donors	9		
Number of H-bond acceptors	8		
Pharmacokinetics properties			
Absorption		Metabolism	
Gastrointestinal absorption	Low	CYP2C9 substrate	No
Hematoencephalic barrier	No	CYP2D6 substrate	No
Caco-2 permeability	Low	CYP3A4 substrate	Yes
p-glicoprotein substrate	Yes	CYP2C9 inhibitor	No
p-glicoprotein inhibitor	No	CYP2D6 Inhibitor	No
Log Kp (skin permeation)	−11.11	CYP3A4 Inhibitor	No
Distribution		CYP1A2 Inhibitor	No
Mitochondrial	Yes	CYP2C19 Inhibitor	No
Plasma protein binding	Low		
Toxicity			
Hepatotoxicity	Inactive	Carcinogenicity	Inactive
Neurotoxicity	Inactive	Immunotoxicity	Active
Nephrotoxicity	Active	Mutagenicity	Inactive
Respiratory toxicity	Activity	Citotoxicity	Inactive
Cardiotoxicity	Inactive		
Predicted acute toxicity in rats (LD_50_)		2.8604 mol/kg	
Predicted human LD_50_		2000 mg/kg	
Expected toxicity class *		IV	

Predictions were obtained from the SwissADME, admetSAR, and PROTOX web servers. * Toxicity classes are defined according to the Globally Harmonized System of Classification and Labeling of Chemicals (GHS). LD50 is expressed in mg/kg. Class I: fatal by ingestion (LD_50_ ≤ 5). Class II: fatal by ingestion (5 < LD_50_ ≤ 50). Class III: fatal by ingestion (50 < LD_50_ ≤ 300). Class IV: fatal by ingestion (300 < LD_50_ ≤ 2000). Class V: fatal by ingestion (2000 < LD_50_ ≤ 5000). Class VI: fatal by ingestion (LD_50_ > 5000).

**Table 5:** Binding energy and interactions of cherimolacyclopeptide D and fidalrestat ligands on the enzyme aldose reductase.

Ligand	Aldose Reductase				
	ΔG (Kcal-mol^−1^)	Ki	H-BR	NPI	RMSD
Cherimolacyclopeptide D	−5.94	43.93 µM	Gln13, Trp307	Trp12, Gln13, Val39, Phe118, Tyr210, and Phe306	-
Fidalrestat	−6.29	24.63 µM	Trp307, Cys301	Trp12, Val39, Tyr40, Trp73, Trp105, Phe118, Tyr210, and Phe306	1.8

**Table 6:** Binding energy and interactions of cherimolacyclopeptide D and metronidazole ligands on the PFOR enzyme.

Ligand	Pyruvate Ferredoxin Oxidoreductase				
	ΔG (Kcal-mol^−1^)	Ki	H-BR	NPI	RMSD
Cherimolacyclopeptide D	−8.2	968.89 nM	Thr31, Asn996, and Lys458	Phe174, His178, Lys435, and Tyr455	-
Metronidazole	−3.28	3.91 nM	Tyr455	Phe174, His178, Lys435, Tyr452, Phe453, and Val431	1.99

## Data Availability

The authors confirm that the data supporting the conclusions of this study are available in the article.

## Supplementary Materials

The following supplementary information can be downloaded at: https://www.mdpi.com/article/10.3390/molecules29235636/s1. Figure S1: ^1^H-NMR spectrum of cherimolacyclopeptide D (**1**) in pyridine-*d*_5_ at 298 K, 700 MHz; Figure S2: ^13^C-NMR spectrum of cherimolacyclopeptide D (**1**) in pyridine-*d*_5_ at 298 K, 175 MHz; Figure S3: HSQC spectrum of cherimolacyclopeptide D (**1**) in pyridine-*d*_5_ at 298 K, 700 MHz; Figure S4: COSY spectrum of cherimolacyclopeptide D (**1**) in pyridine-*d*_5_ at 298 K, 700 MHz; Figure S5: TOCSY spectrum of cherimolacyclopeptide D (**1**) in pyridine-*d*_5_ at 298 K, 700 MHz; Figure S6: HMBC spectrum of cherimolacyclopeptide D (**1**) in pyridine-*d*_5_ at 298 K, 700 MHz; Figure S7: MALDI-TOF mass spectrum of cherimolacyclopeptide D (**1**) *m*/*z*: of 675 [M + Na]^+^; Figure S8: IR spectrum of cherimolacyclopeptide D (**1**); Figure S9: ^1^H-NMR spectrum of squamin D (**2**) in acetone-*d*_6_ at 298 K, 700 MHz; Figure S10: ^1^H-NMR spectrum of squamin D (**2**) in CD_3_OD at 298 K, 400 MHz; Figure S11: ^13^C-NMR spectrum of squamin D (**2**) in acetone-*d*_6_ at 298 K, 175 MHz; Figure S12: HSQC spectrum of squamin D (**2**) in acetone-*d*_6_ at 298 K, 700 MHz; Figure S13: COSY spectrum of squamin D (**2**) in acetone-*d*_6_ at 298 K, 700 MHz; Figure S14: TOCSY spectrum of squamin D (**2**) in acetone-*d*_6_ at 298 K, 700 MHz; Figure S15: HMBC spectrum of squamin D (**2**) in acetone-*d*_6_ at 298 K, 700 MHz; Figure S16: MALDI-TOF mass spectrum of squamin D (**2**) with *m*/*z*: of 843 [M + Na]^+^; Figure S17: ^1^H-NMR spectrum of squamin C (**3**) in acetone-*d*_6_ at 298 K, 700 MHz; Figure S18: ^1^H-NMR spectrum of squamin C (**3**) in CD_3_OD at 298 K, 400 MHz; Figure S19: ^13^C-NMR spectrum of squamin C (**3**) in acetone-*d*_6_ at 298 K, 175 MHz; Figure S20: HSQC spectrum of squamin C (**3**) in acetone-*d*_6_ at 298 K, 700 MHz; Figure S21: COSY spectrum of squamin C (**3**) in acetone-*d*_6_ at 298 K, 700 MHz; Figure S22: TOCSY spectrum of squamin C (**3**) in acetone-*d*_6_ at 298 K, 700 MHz; Figure S23: HMBC spectrum of squamin C (**3**) in acetone-*d*_6_ at 298 K, 700 MHz; Figure S24: MALDI-TOF mass spectrum of squamin C (**3**) with *m*/*z*: of 843 [M + Na]^+^.
