# Supplementary material for: Antiprotozoal Activity Against Entamoeba hystolitica and Giardia lamblia of Cyclopeptides Isolated from Annona diversifolia Saff
Source: Molecules. 2024 Nov 28;29(23):5636. doi: 10.3390/molecules29235636 (PMC11643917; doi:10.3390/molecules29235636)
Supplement: Supplementary file 1 [file molecules-29-05636-s001.zip › molecules-3311066-supplementary.pdf]

Antiprotozoal activity against *Entamoeba hystolitica* and *Giardia lamblia* of cyclopeptides isolated from *Annona diversifolia* Saff.

Ulises Murrieta-Dionicio [1], Fernando Calzada [2*], Elizabeth Barbosa [3], Miguel Valdés [3], Benito Reyes-Trejo [1*], Holber Zuleta-Prada [1], Diana Guerra-Ramírez [1], and Federico del Río-Portilla [4]

[1] Laboratorio de Productos Naturales, Área de Química, Departamento de Preparatoria Agrícola, Universidad Autónoma Chapingo, km 38.5 Carretera México-Texcoco, Chapingo 56230, México; murrieta.dionicio.091293@gmail.com (U.M.-D.); breyest@chapingo.mx (B.R-T.); dguerrar@chapingo.mx (D.G-R.); hzuletap@chapingo.mx (H.Z-P.)

[2] Unidad de Investigación Médica en Farmacología, UMAE Hospital de Especialidades, 2° Piso CORSE Centro Médico Nacional Siglo XXI, IMSS, Av. Cuauhtémoc 330, Col. Doctores, Ciudad de México CP 06725, México; fernando.calzada@imss.gob.mx o fercalber10@gmail.com (F.C.); valdesguevarami-guel@gmail.com (M.V.)

[3] Escuela Superior de Medicina, Instituto Politécnico Nacional, Salvador Díaz Mirón esq. Plan de San Luis S/N, Miguel Hidalgo, Casco de Santo Tomas, Ciudad de México CP 11340, México; rebc78@yahoo.com.mx (E.B.)

[4] Instituto de Química, Universidad Nacional Autónoma de México, Ciudad Universitaria, C.P. 04510, México; jfrp@unam.mx (F.d.-R.-P.)

*Authors to whom correspondence should be addressed; E-Mails: breyest@chapingo.mx (B.R.T.); fercalber10@gmail.com/fernando.calzada@imss.gob.mx (F.C.),

Support Information

Figure S1. ^{1}H-NMR spectrum of cherimolacyclopeptide D (**1**) in pyridine-d_5 at 298 K, 700 MHz. 3
Figure S2. ^{13}C-NMR spectrum of cherimolacyclopeptide D (**1**) in pyridine-d_5 at 298 K, 175 MHz..... 3
Figure S3. HSQC spectrum of cherimolacyclopeptide D (**1**) in pyridine-d_5 at 298 K, 700 MHz......... 4
Figure S4. COSY spectrum of cherimolacyclopeptide D (**1**) in pyridine-d_5 at 298 K, 700 MHz. 5
Figure S5. TOCSY spectrum of cherimolacyclopeptide D (**1**) in pyridine-d_5 at 298 K, 700 MHz...... 6
Figure S6. HMBC spectrum of cherimolacyclopeptide D (**1**) in pyridine-d_5 at 298 K, 700 MHz....... 7
Figure S7. MALDI-TOF mass spectrum of cherimolacyclopeptide D (**1**) m/z: of 675 $[M+Na]^+$........ 8
Figure S8. IR spectrum of cherimolacyclopeptide D (**1**). 8
Figure S9. ^{1}H-NMR spectrum of squamin D (**2**) in acetone-d_6 at 298 K, 700 MHz. 9
Figure S10. ^{1}H-NMR spectrum of squamin D (**2**) in CD_3OD at 298 K, 400 MHz. 9
Figure S11. ^{13}C-NMR spectrum of squamin D (**2**) in acetone-d_6 at 298 K, 175 MHz. 10
Figure S12. HSQC spectrum of squamin D (**2**) in acetone-d_6 at 298 K, 700 MHz. 11
Figure S13. COSY spectrum of squamin D (**2**) in acetone-d_6 at 298 K, 700 MHz. 12
Figure S14. TOCSY spectrum of squamin D (**2**) in acetone-d_6 at 298 K, 700 MHz. 13
Figure S15. HMBC spectrum of squamin D (**2**) in acetone-d_6 at 298 K, 700 MHz. 14
Figure S16. MALDI-TOF mass spectrum of squamin D (**2**) with m/z: of 843 $[M+Na]^+$. 15
Figure S17. ^{1}H-NMR spectrum of squamin C (**3**) in acetone-d_6 at 298 K, 700 MHz.......................... 16
Figure S18. ^{1}H-NMR spectrum of squamin C (**3**) in CD_3OD at 298 K, 400 MHz. 16
Figure S19. ^{13}C-NMR spectrum of squamin C (**3**) in acetone-d_6 at 298 K, 175 MHz. 17
Figure S20. HSQC spectrum of squamin C (**3**) in acetone-d_6 at 298 K, 700 MHz............................ 18
Figure S21. COSY spectrum of squamin C (**3**) in acetone-d_6 at 298 K, 700 MHz............................ 19
Figure S22. TOCSY spectrum of squamin C (**3**) in acetone-d_6 at 298 K, 700 MHz. 20
Figure S23. HMBC spectrum of squamin C (**3**) in acetone-d_6 at 298 K, 700 MHz........................... 21
Figure S24. MALDI-TOF mass spectrum of squamin C (**3**) with m/z: of 843 $[M+Na]^+$.................. 22

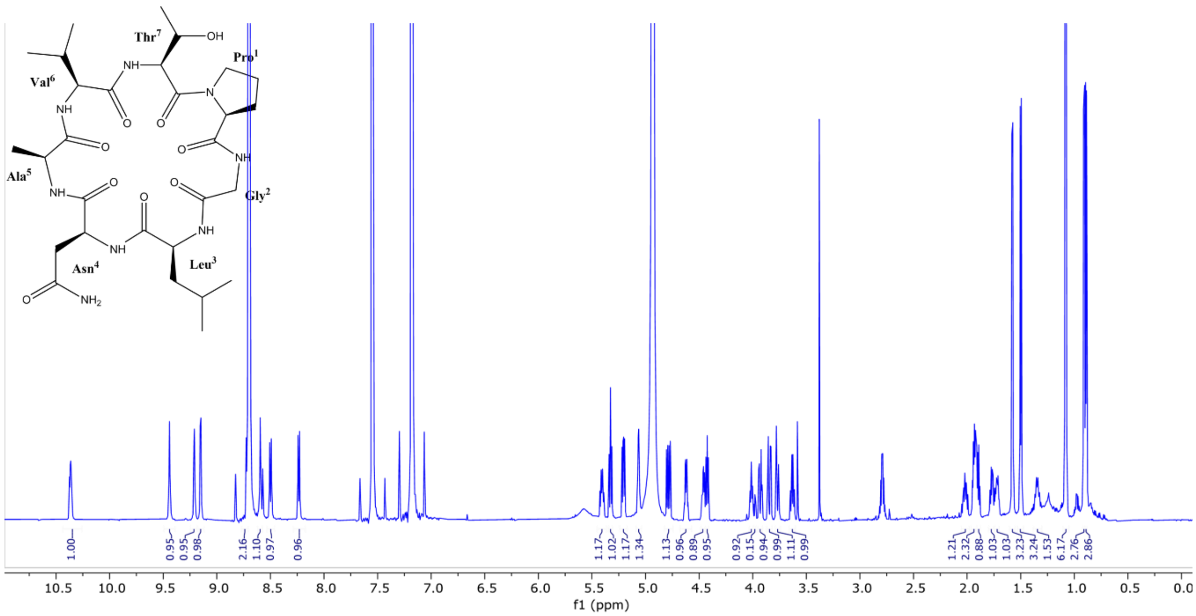

Figure S1. ^{1}H-NMR spectrum of cherimolacyclopeptide D (**1**) in pyridine-d_5 at 298 K, 700 MHz.

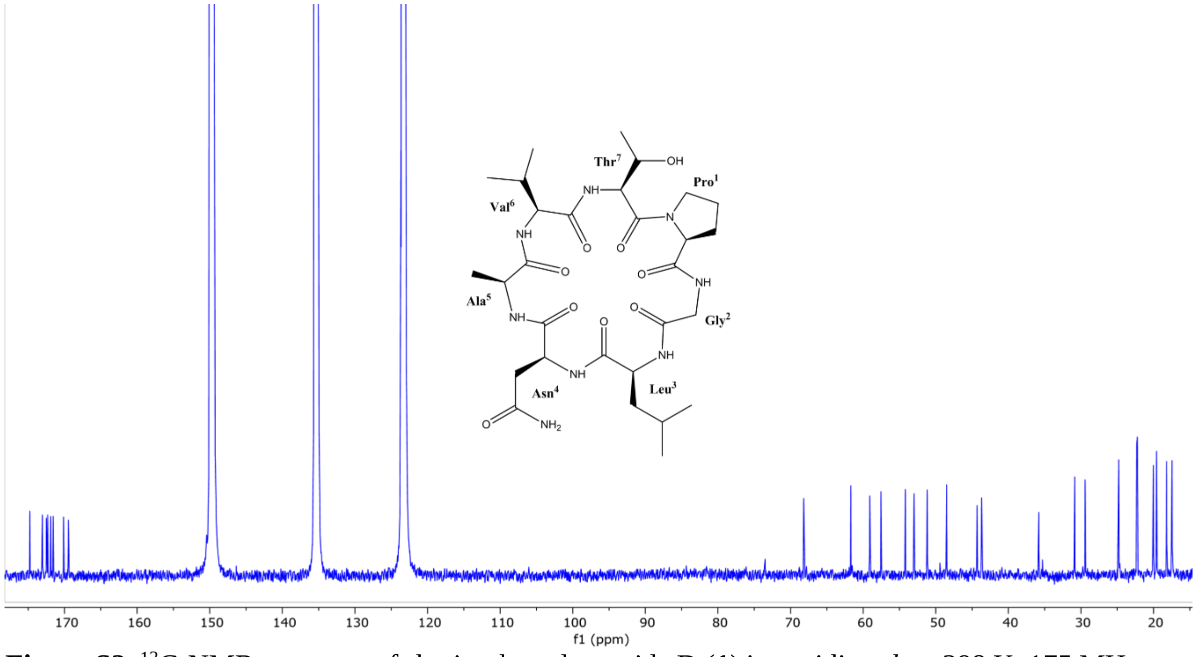

Figure S2. ^{13}C-NMR spectrum of cherimolacyclopeptide D (**1**) in pyridine-d_5 at 298 K, 175 MHz.

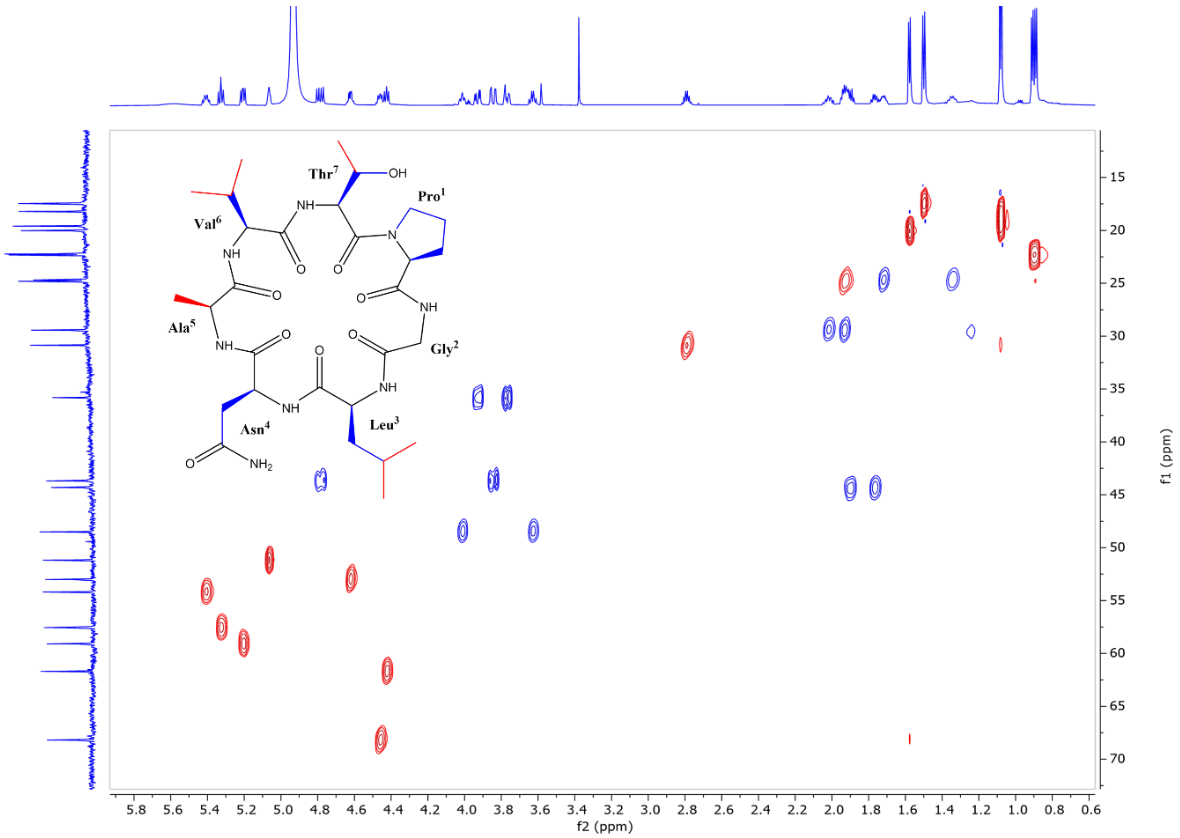

Figure S3. HSQC spectrum of cherimolacyclopeptide D (**1**) in pyridine-d_5 at 298 K, 700 MHz.

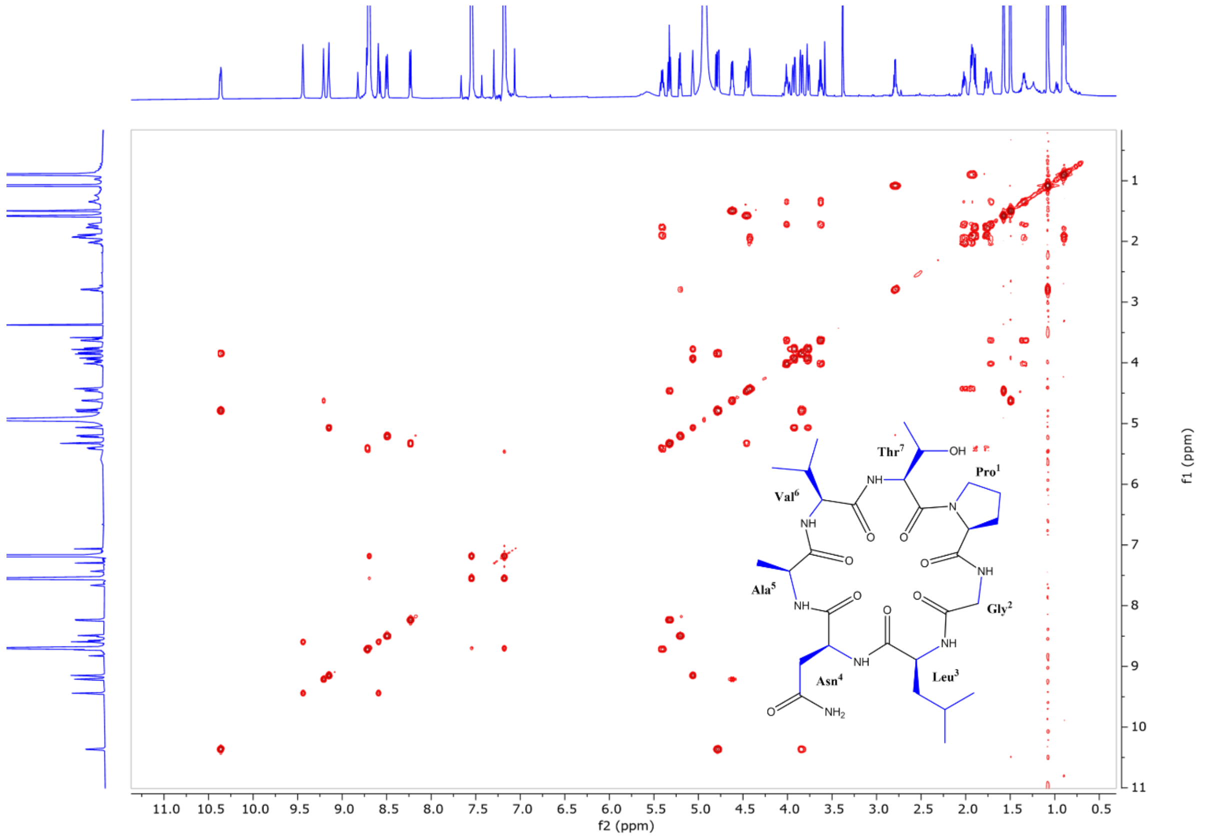

Figure S4. COSY spectrum of cherimolacyclopeptide D (**1**) in pyridine-d_5 at 298 K, 700 MHz.

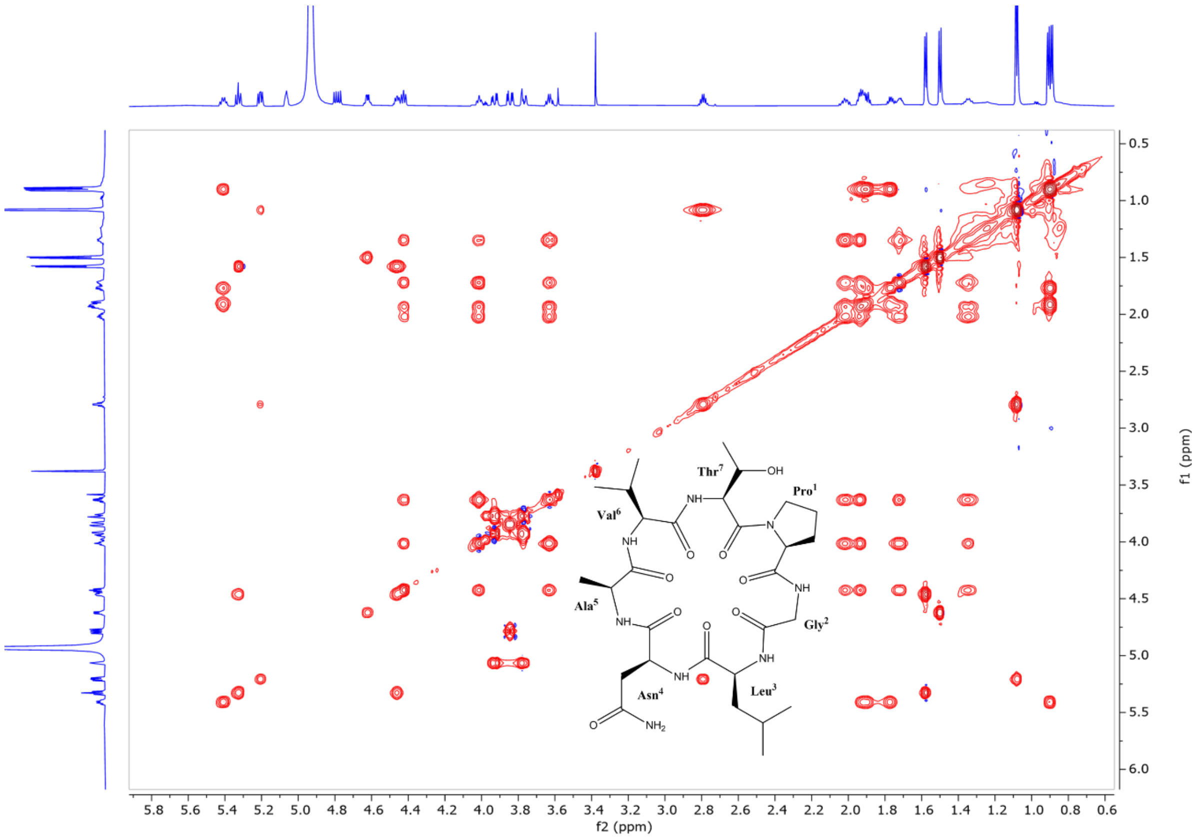

Figure S5. TOCSY spectrum of cherimolacyclopeptide D (**1**) in pyridine-d_5 at 298 K, 700 MHz.

.

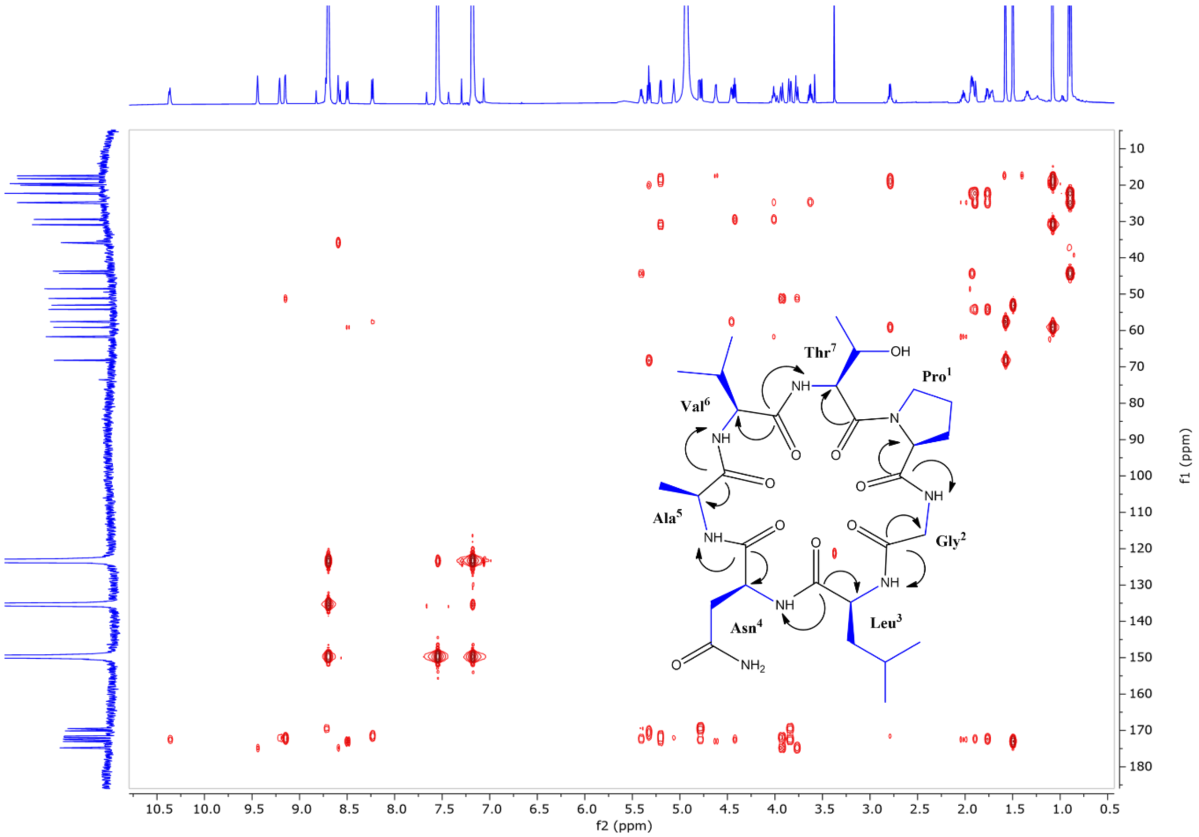

Figure S6. HMBC spectrum of cherimolacyclopeptide D (**1**) in pyridine-d_5 at 298 K, 700 MHz.

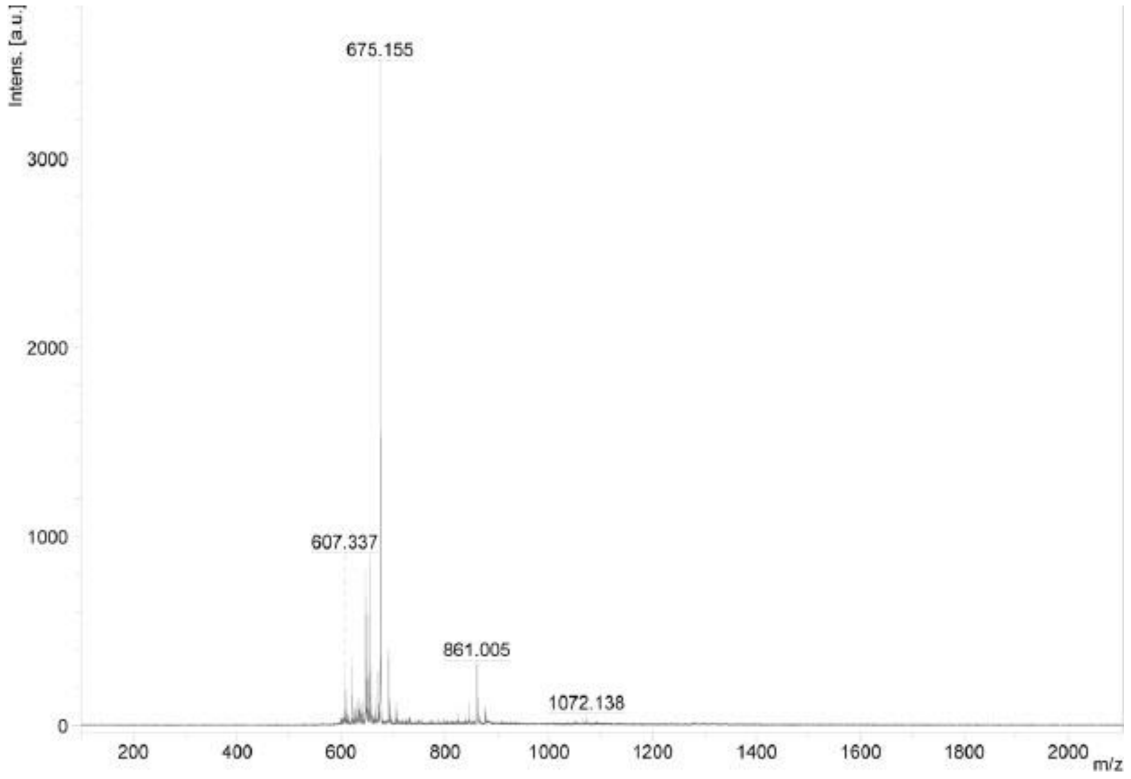

Figure S7. MALDI-TOF mass spectrum of cherimolacyclopeptide D (**1**) m/z: of 675 [M+Na]$^{+}$.

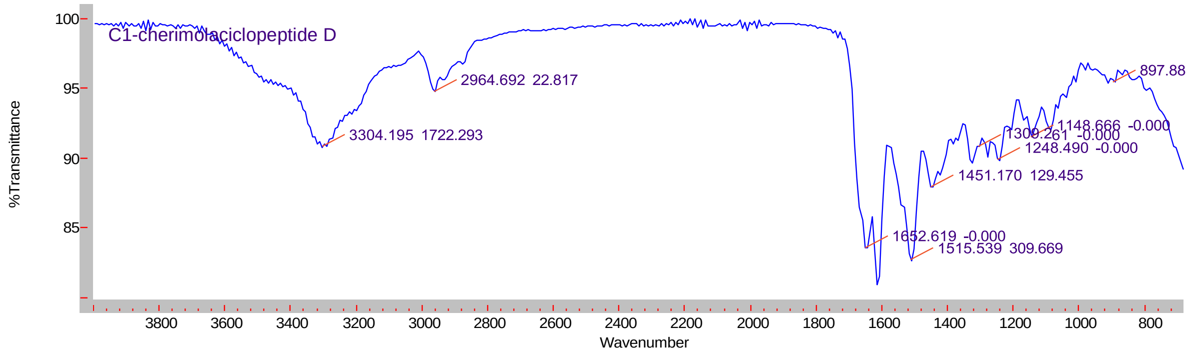

Figure S8. IR spectrum of cherimolacyclopeptide D (**1**).

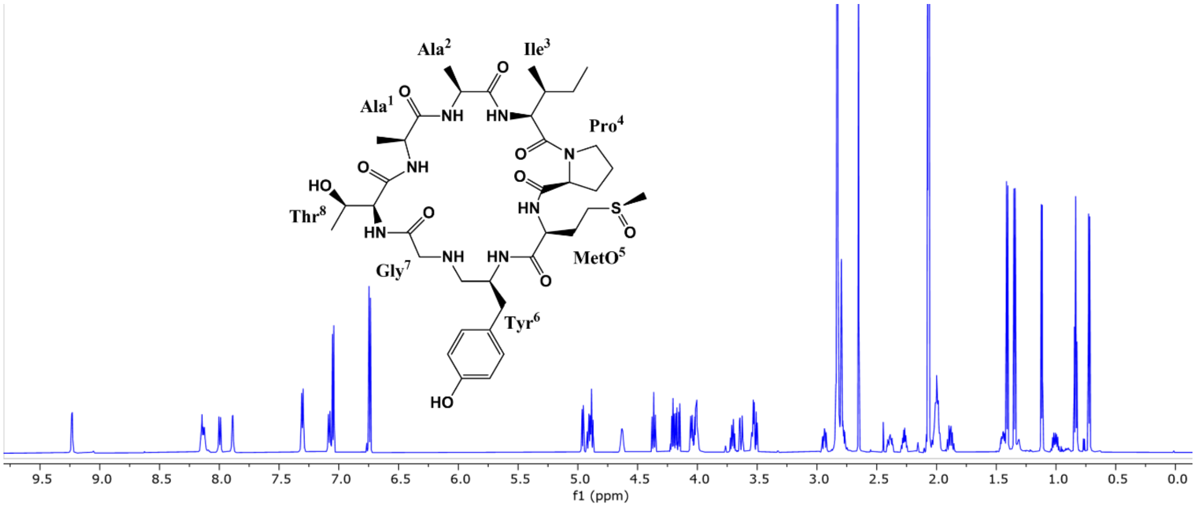

Figure S9. ^{1}H-NMR spectrum of squamin D (**2**) in acetone-d_6 at 298 K, 700 MHz.

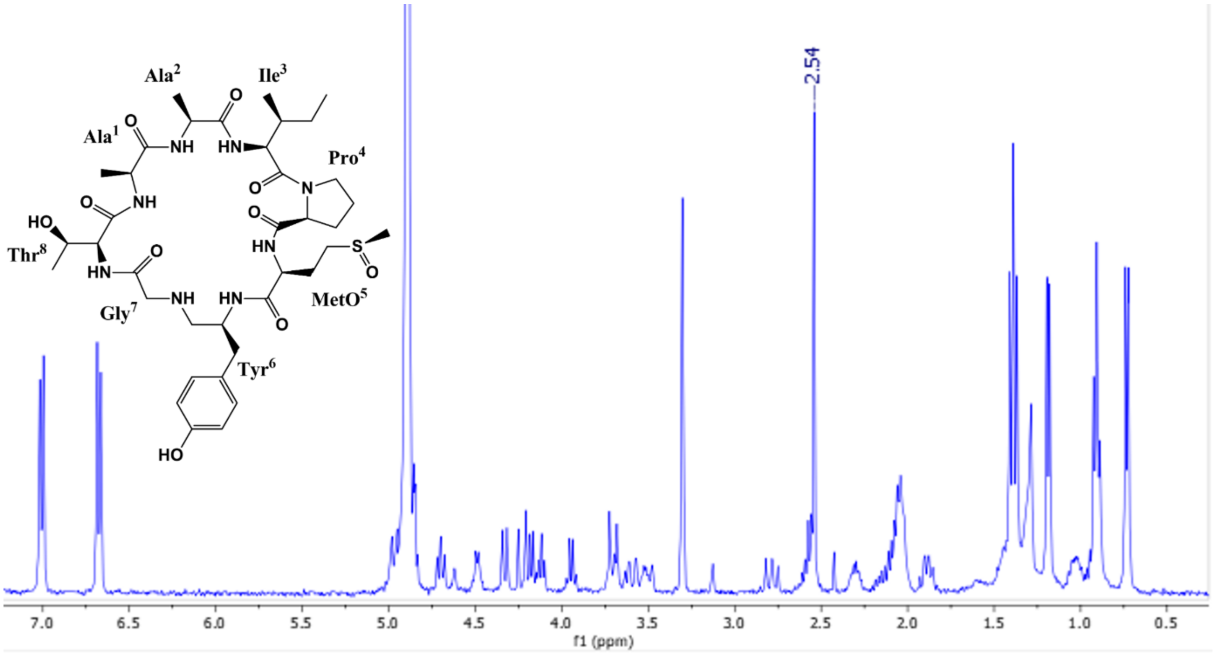

Figure S10. ^{1}H-NMR spectrum of squamin D (**2**) in CD_3OD at 298 K, 400 MHz.

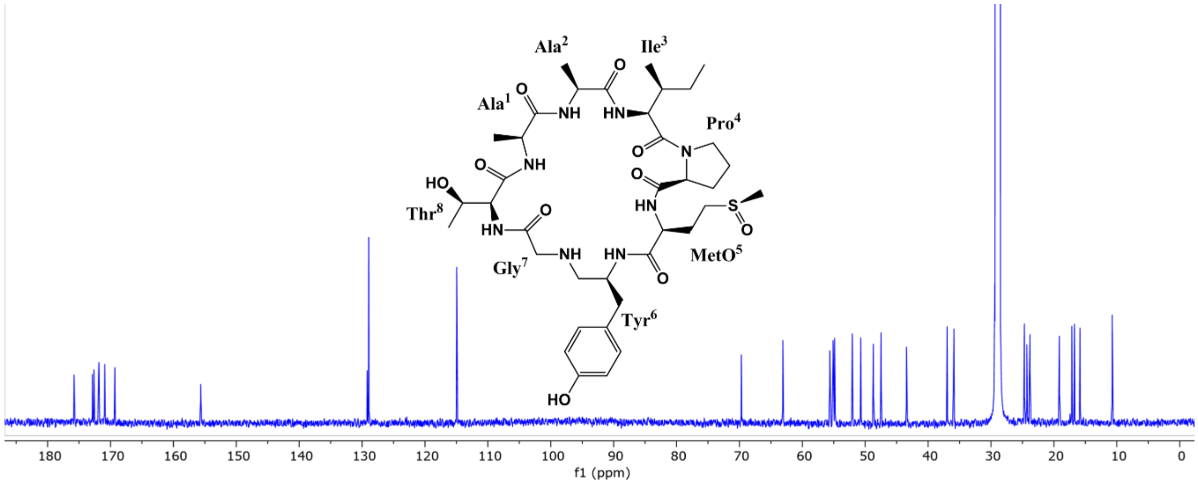

Figure S11. ^{13}C-NMR spectrum of squamin D (**2**) in acetone-d_6 at 298 K, 175 MHz.

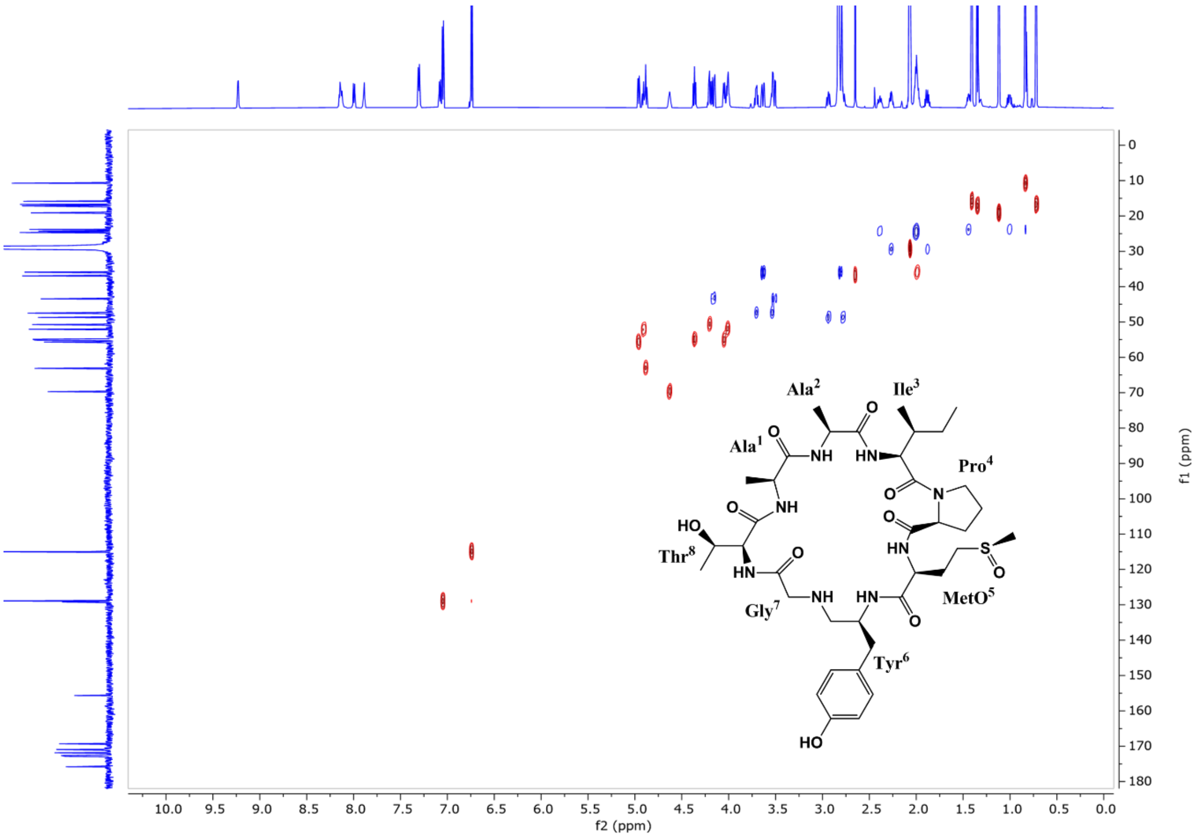

Figure S12. HSQC spectrum of squamin D (**2**) in acetone-d_6 at 298 K, 700 MHz.

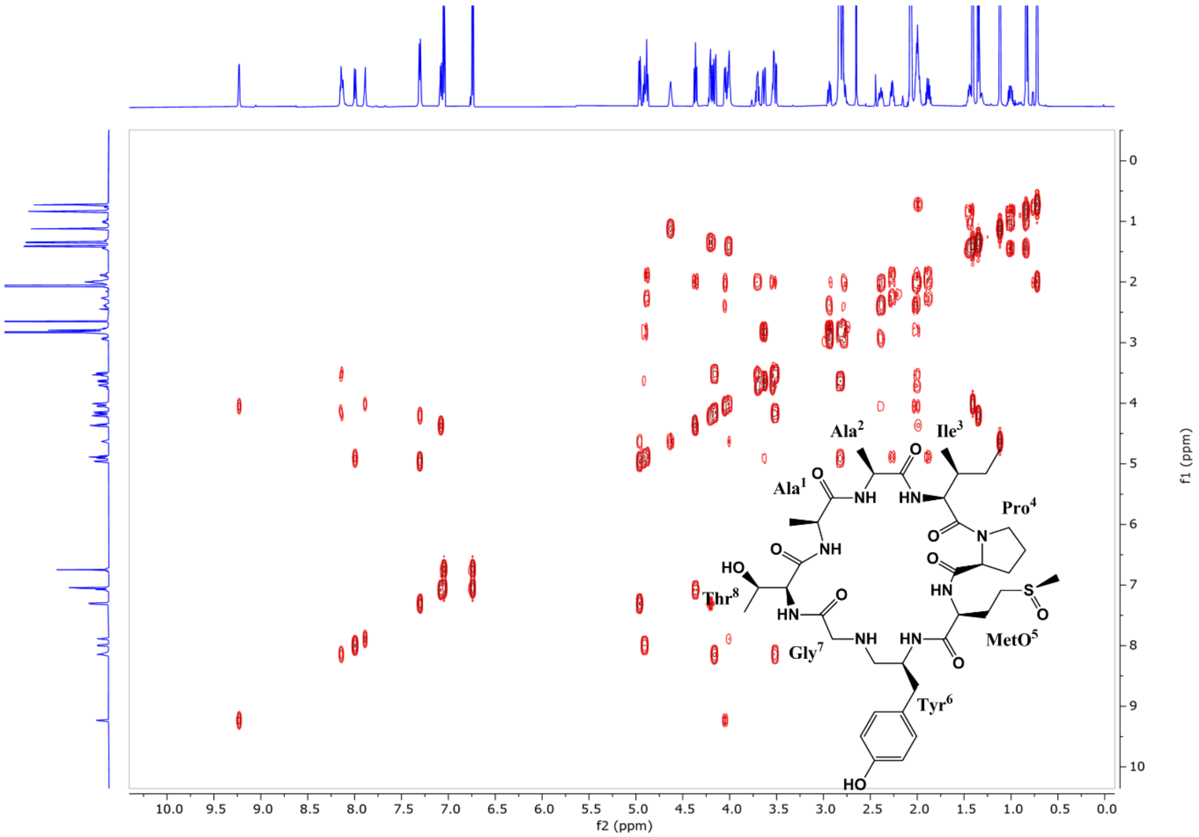

Figure S13. COSY spectrum of squamin D (**2**) in acetone-d_6 at 298 K, 700 MHz.

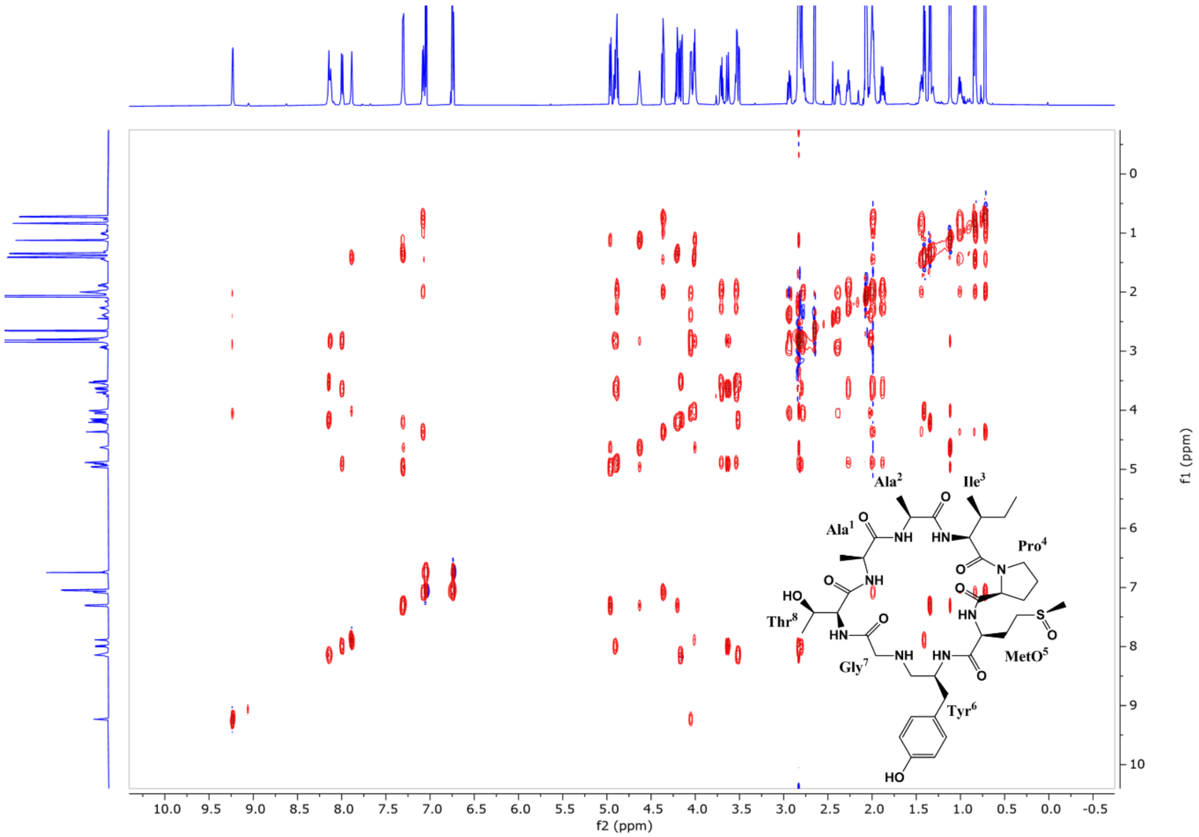

Figure S14. TOCSY spectrum of squamin D (**2**) in acetone-d_6 at 298 K, 700 MHz.

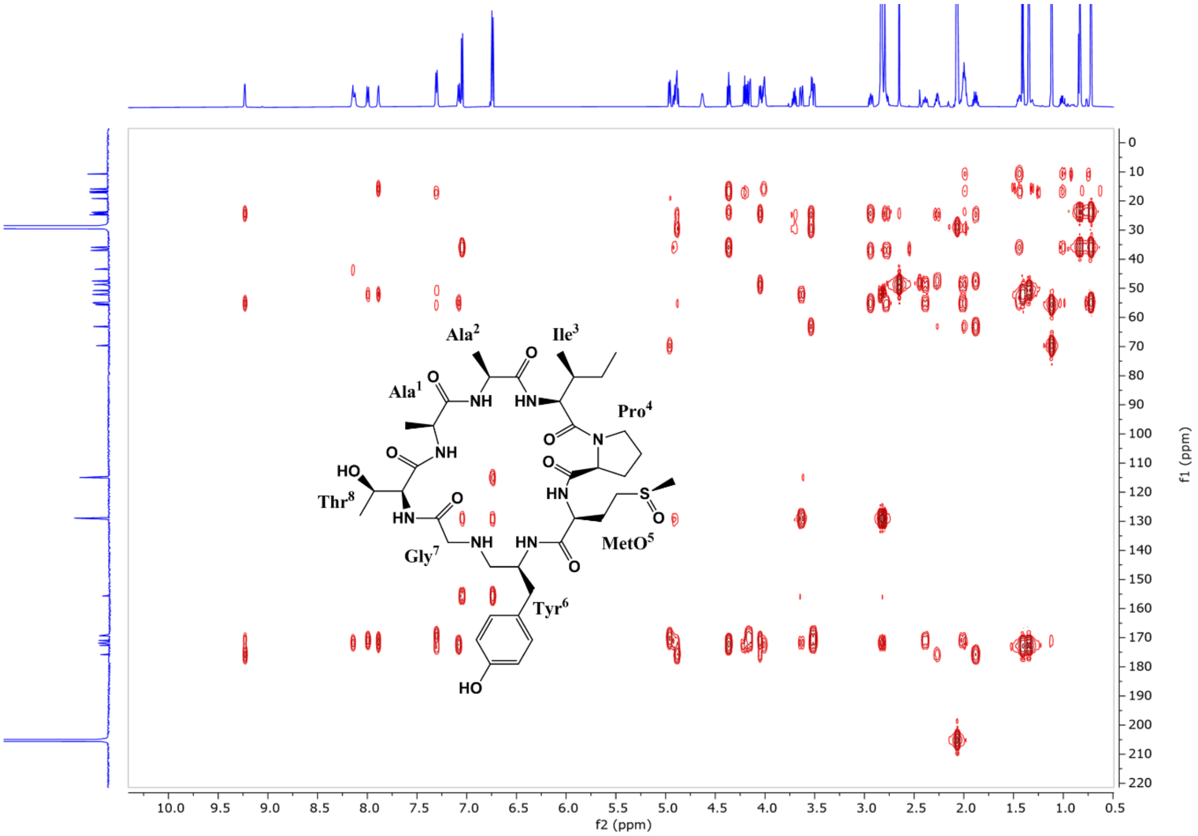

Figure S15. HMBC spectrum of squamin D (**2**) in acetone-d_6 at 298 K, 700 MHz.

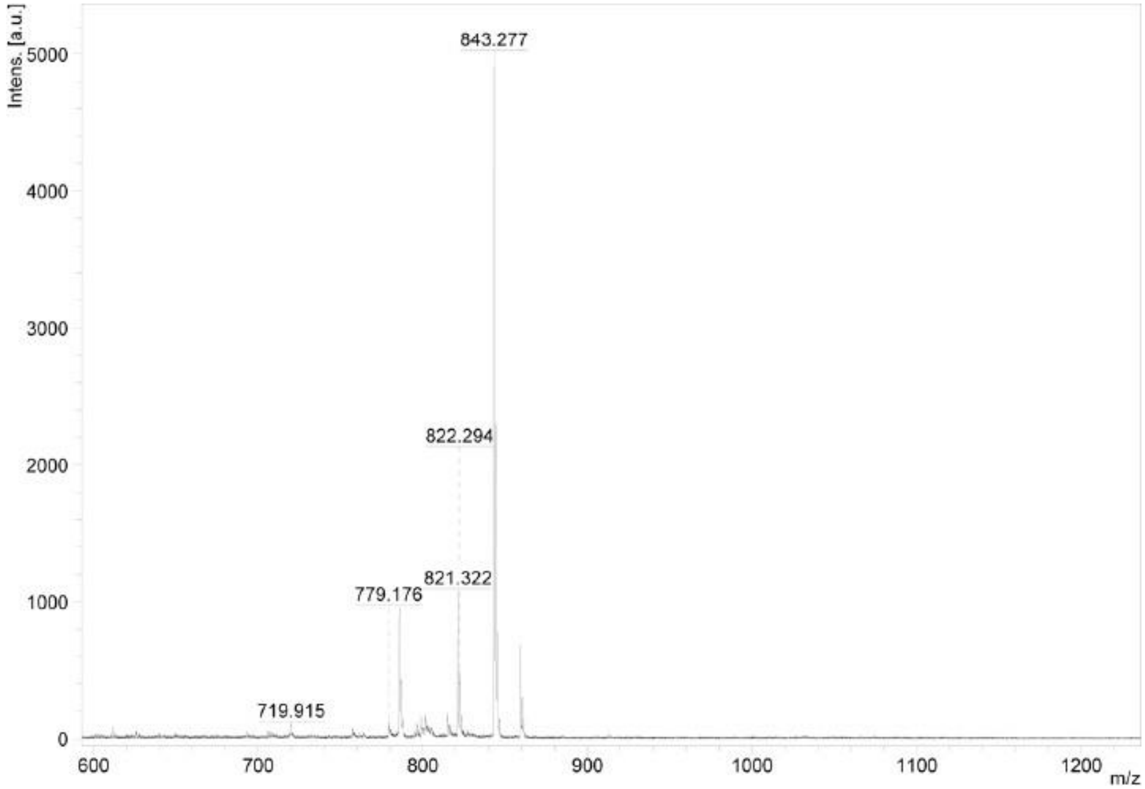

Figure S16. MALDI-TOF mass spectrum of squamin D (**2**) with m/z: of 843 $[M+Na]^+$.

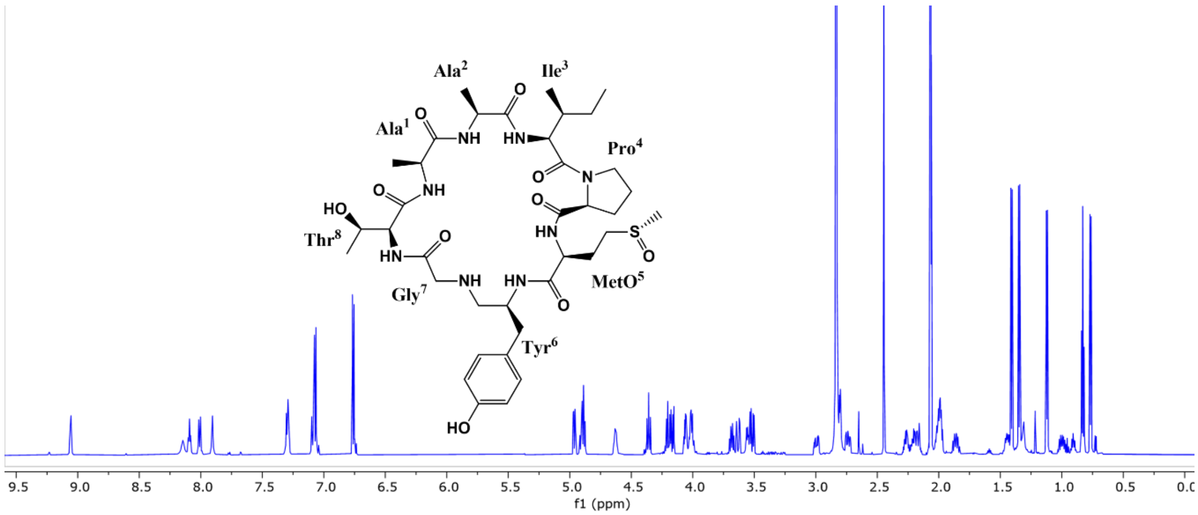

Figure S17. ^{1}H-NMR spectrum of squamin C (**3**) in acetone-d_6 at 298 K, 700 MHz.

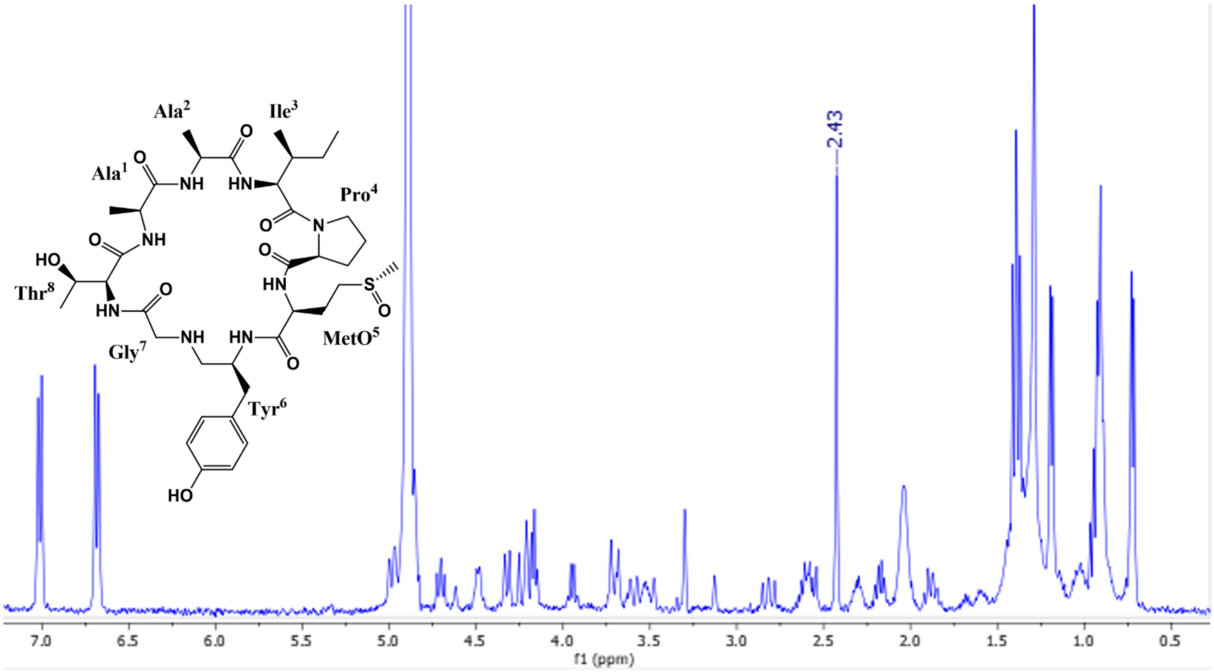

Figure S18. ^{1}H-NMR spectrum of squamin C (**3**) in CD_3OD at 298 K, 400 MHz.

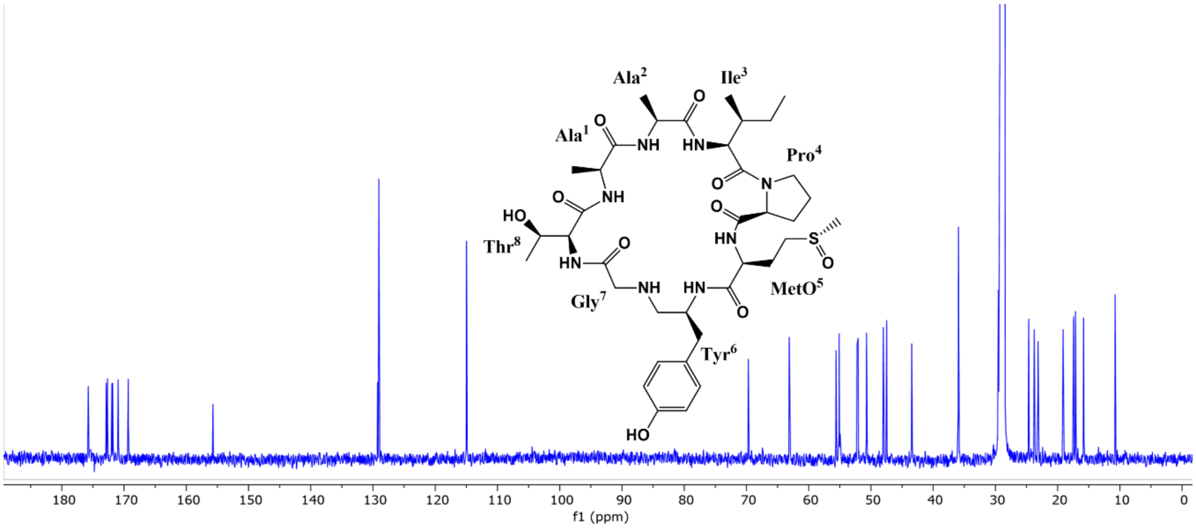

Figure S19. ^{13}C-NMR spectrum of squamin C (**3**) in acetone-d_6 at 298 K, 175 MHz.

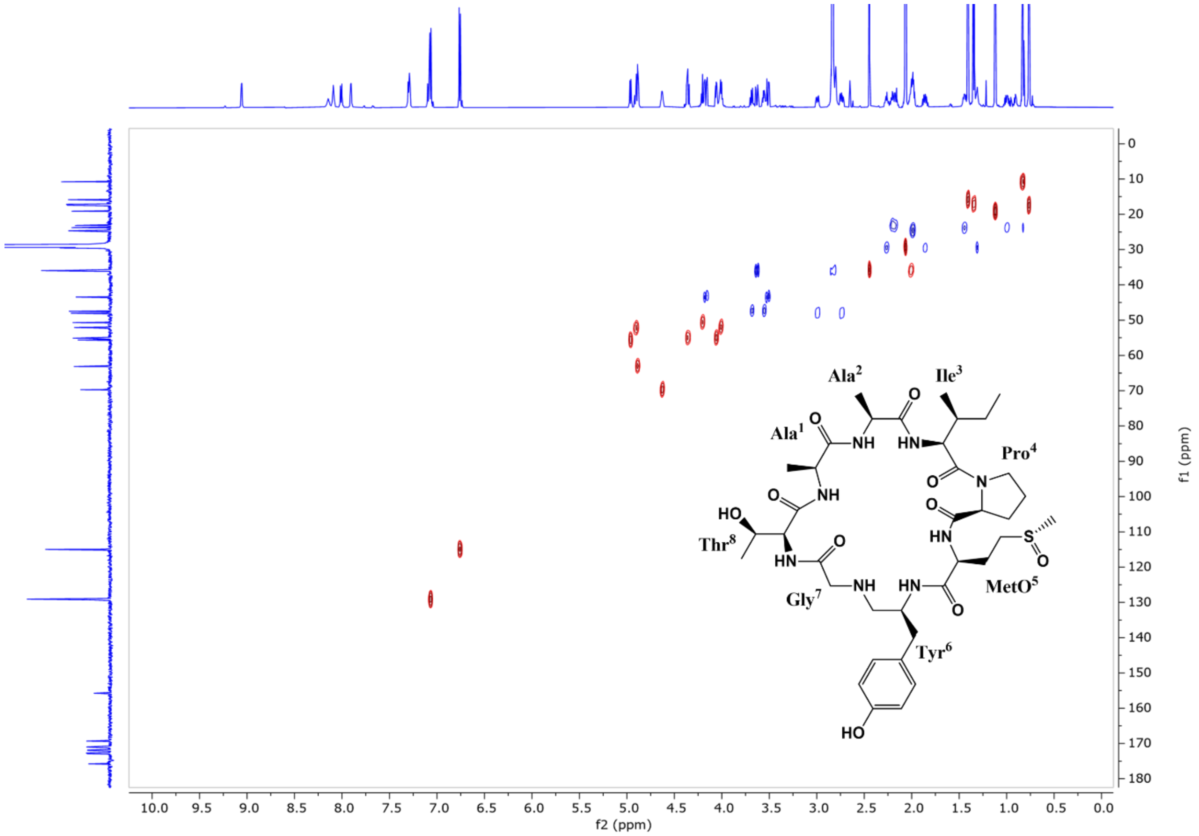

Figure S20. HSQC spectrum of squamin C (**3**) in acetone-d_6 at 298 K, 700 MHz.

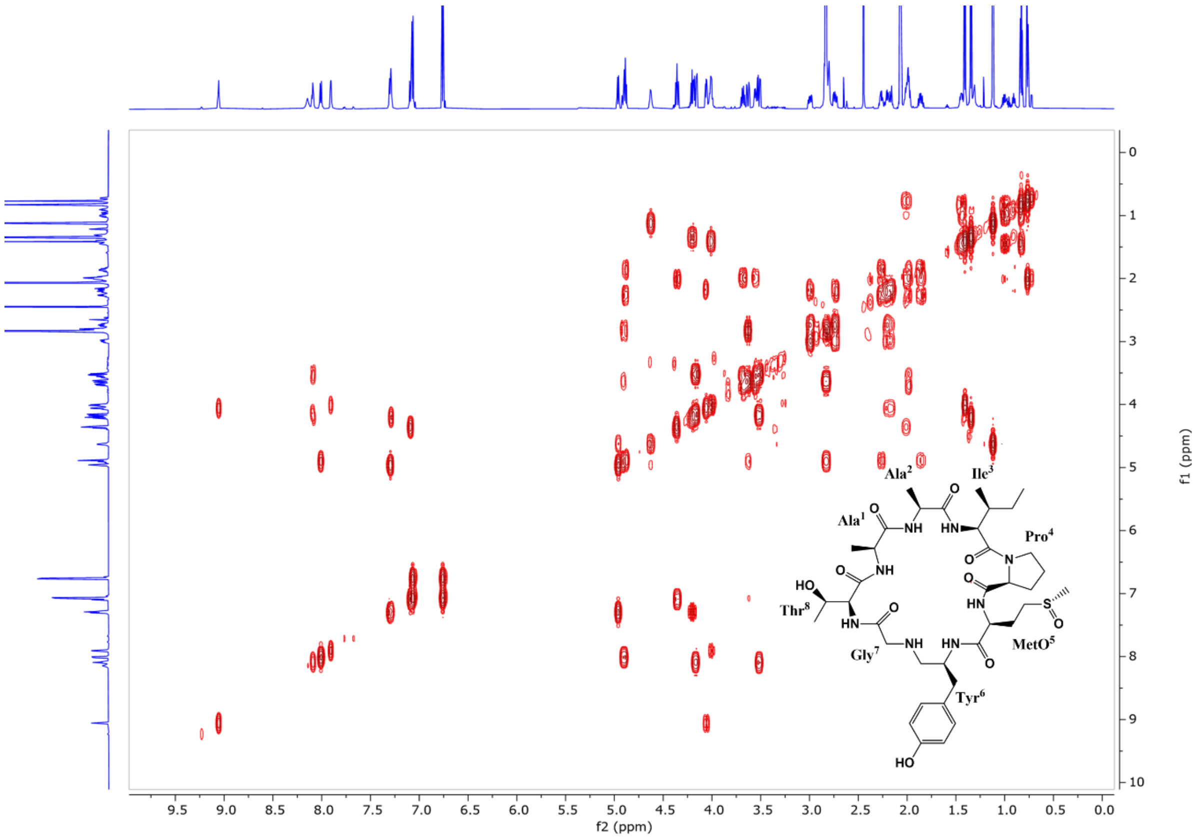

Figure S21. COSY spectrum of squamin C (**3**) in acetone-d_6 at 298 K, 700 MHz.

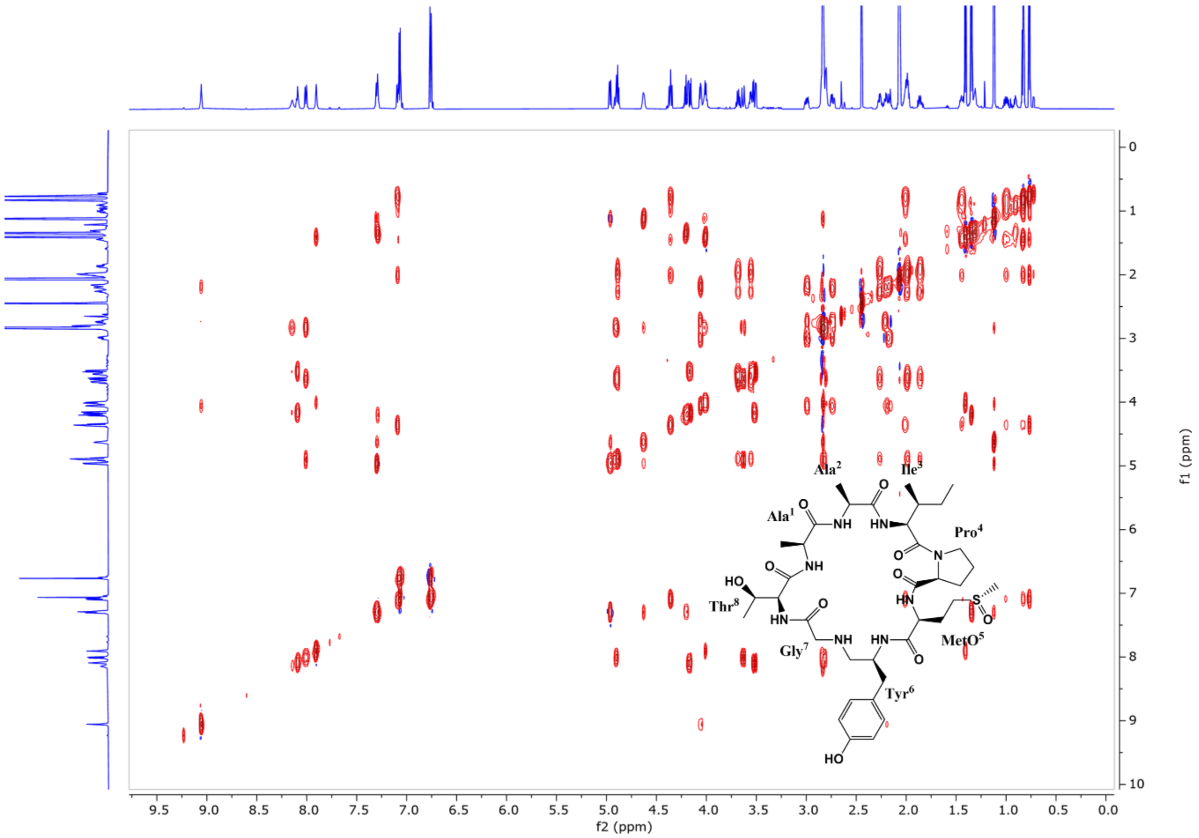

Figure S22. TOCSY spectrum of squamin C (**3**) in acetone-d_6 at 298 K, 700 MHz.

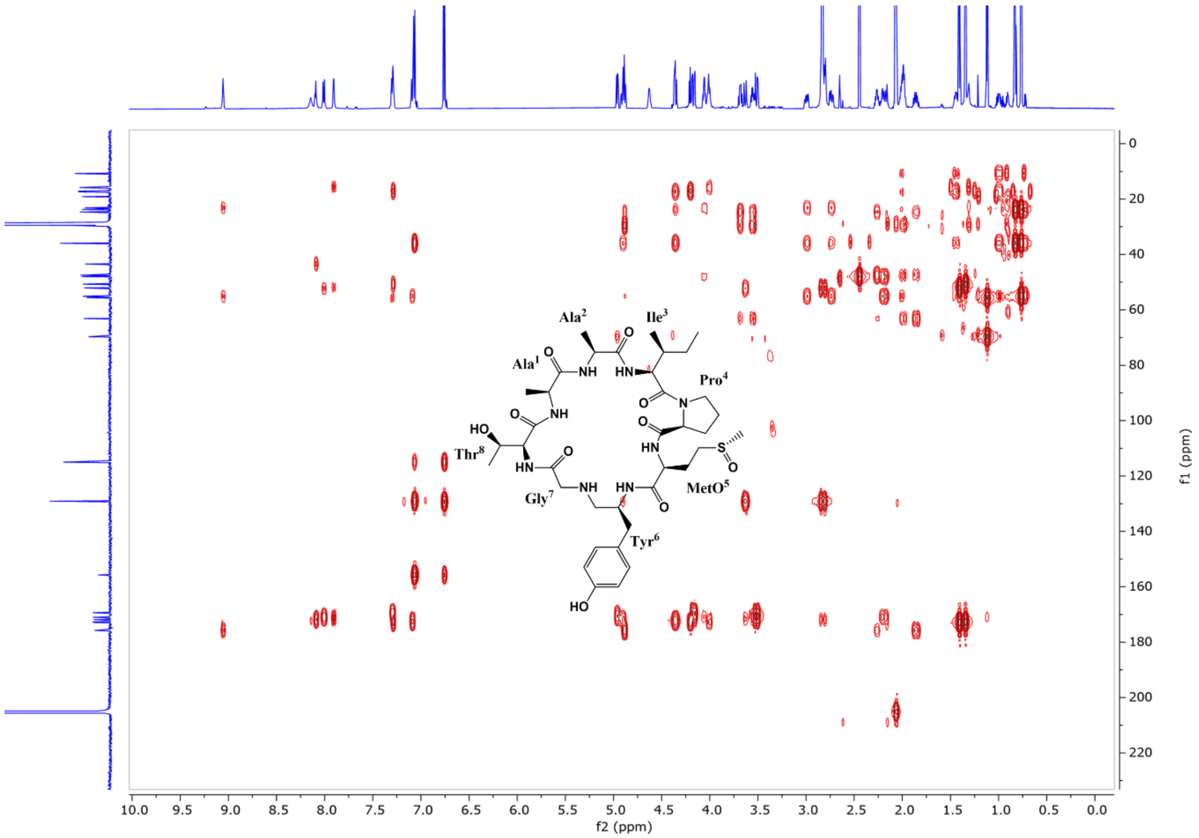

Figure S23. HMBC spectrum of squamin C (**3**) in acetone-d_6 at 298 K, 700 MHz.

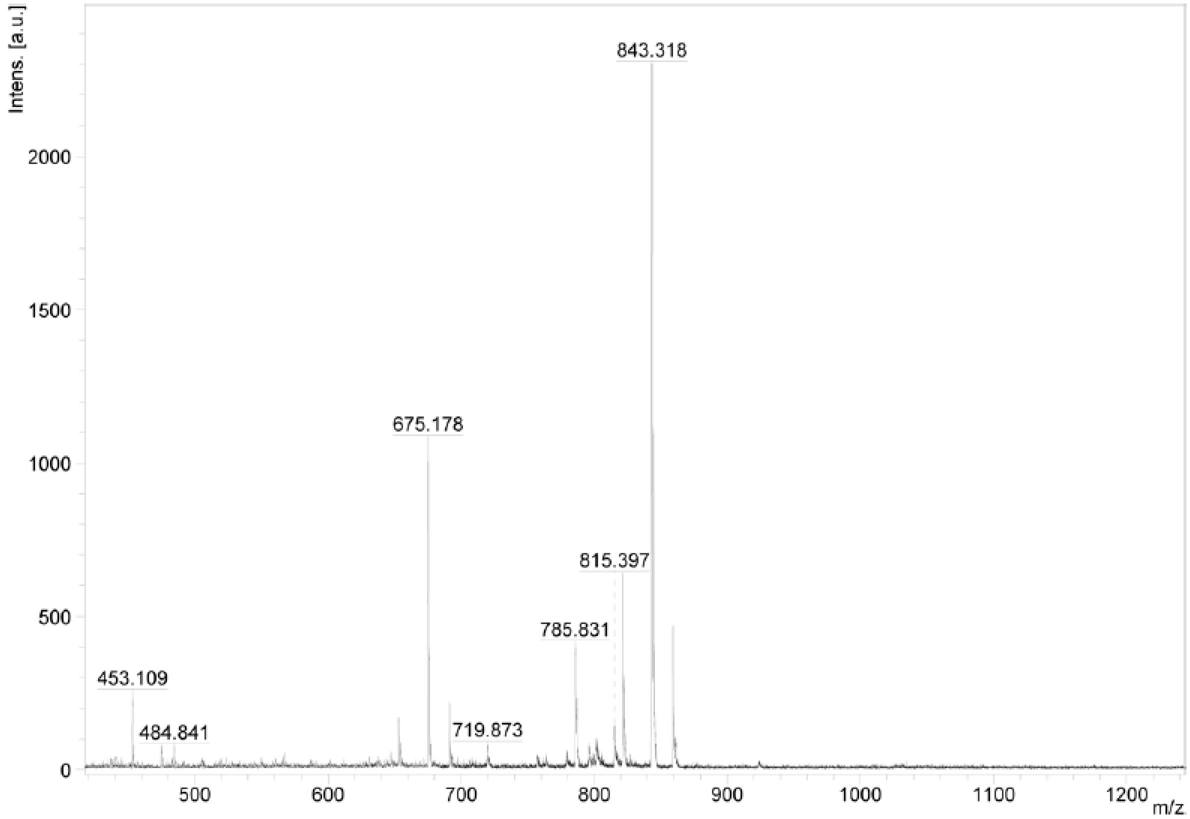

Figure S24. MALDI-TOF mass spectrum of squamin C (**3**) with m/z: of 843 $[M+Na]^+$.
